# Engaging rural communities in Bangladesh to address antimicrobial resistance via the community dialogue approach: a protocol for a cluster-randomized controlled trial

**DOI:** 10.3389/fpubh.2025.1432635

**Published:** 2025-12-17

**Authors:** Rebecca King, Joseph Paul Hicks, Fariza Fieroze, Md. Badruddin Saify, S. M. Abdullah, Dani Barrington, Prudence Hamade, Helen Hawkings, Tim Ensor, Sophia Latham, Jessica Mitchell, Amam Zonaed Siddiki, Rumana Huque

**Affiliations:** 1 Nuffield Centre for International Health and Development, Leeds Institute for Health Sciences, University of Leeds, Leeds, United Kingdom; 2 ARK Foundation, Dhaka, Bangladesh; 3 School of Population and Global Health, The University of Western Australia, Crawley, WA, Australia; 4 Malaria Consortium, London, United Kingdom; 5 Department of Livestock and One Health, Institute of Veterinary and Ecological Sciences, University of Liverpool, Neston, United Kingdom; 6 Faculty of Veterinary Medicine, Chattogram Veterinary and Animal Sciences University, Chattogram, Bangladesh

**Keywords:** antibiotic resistance, antimicrobial resistance, community engagement, community education, low- and middle-income countries, Bangladesh

## Abstract

**Introduction:**

To effectively tackle antibiotic resistance (ABR) a One Health approach is required, focusing on the human, animal and environmental sectors together, and that public education and engagement programs must be part of the overall approach. However, there has been limited research on such programs in low−/middle-income countries (LMICs). Here we describe our plans to evaluate a community-engagement program, known as the community dialogue approach, that takes a One Health approach to tackling ABR in rural communities in Bangladesh, and involves community-led and community-based education and discussion forums. Members of our team previously developed this approach and used it to address other health issues in other LMIC contexts, while our team has previously adapted it for this topic and setting.

**Methods:**

We will use a pragmatic, non-blinded, two-arm, parallel-group, cluster-randomized, controlled trial to primarily evaluate whether the intervention can improve (1) the level of correct and appropriate knowledge about antibiotics, ABR, and antibiotic usage from a One Health perspective, (2) levels of awareness about the existence of antibiotics and ABR, and (3) the relative frequency of self-reported and observable indicators of best practices related to antibiotic usage. Within Cumilla district, we will randomize 50 clusters of villages in a 1:1 ratio. In intervention community clusters trained community volunteers will deliver a set of 11 health education and discussion forums across a 12-month period, while control community clusters will receive no inputs. We will collect outcomes at baseline (pre-randomization) and endline (following the final community dialogue) via two repeated cross-sectional household surveys (each aiming to survey 2,200 participants across all clusters). We will also conduct nested process evaluation and costing studies.

**Discussion:**

Community engagement approaches have successfully addressed other health issues in low resource settings, but there is limited evidence on using community engagement approaches to address ABR in low resource contexts, particularly in Bangladesh. We will closely involve the Bangladeshi health system in this research to ensure feasibility and facilitate scale-up via an embedded approach.

**Clinical trial registration:**

https://doi.org/10.1186/ISRCTN93756764, identifier ISRCTN93756764.

## Introduction

### Background and rationale

Antimicrobial resistance (AMR) is now widely considered to be one of the most important global challenges to public health and development (1). Accurate yearly estimates of global mortality due to antimicrobial resistant infections are not available, but one recent attempt estimated that 4.95 million deaths were associated with bacterial AMR in 2019 (2). Similarly, while highly speculative, a recent estimate under a “low-AMR scenario” predicted annual global costs of $1 trillion annually due to the impacts of AMR from 2030, increasing to $2 trillion annually by 2050 (3). Global efforts to tackle AMR have increasingly emphasized the need to take a One Health approach: recognizing that human, animal and environmental health are all interconnected, and that to successfully tackle AMR we must address the different but interlinked causes of AMR operating within these sectors simultaneously. This is reflected in the 2016 United Nations Political Declaration on AMR (1) and other international action plans, such as the World Health Organization’s (WHO) 2015 Global Action Plan on Antimicrobial Resistance (4).

Low- and middle-income countries (LMICs) are already being more severely impacted by the effects of AMR compared to high-income countries, and this situation is only expected to worsen in the future (2). However, it is not currently possible to robustly identify the different possible causal factors and their relative contributions to the various negative impacts of AMR, given the scale and complexity of the issue. Within LMICs among the factors most often proposed as likely key causes of rising levels of AMR are the sometimes high levels of antimicrobial misuse that occur when treating human and animal health issues (i.e., the prescription or sale of inappropriate and/or unneeded antimicrobials), and in the case of animal husbandry the increasing use of antimicrobials as prophylactics and growth promoters (as has been routinely the case in high income countries for many decades) (5–7). In some LMICs this misuse is facilitated by the easy and unregulated public access to antimicrobials (8), a lack of education and awareness about AMR and its negative impacts (9), and unhelpful cultural beliefs and practices related to medicines and their use (5, 6). Practically speaking, these issues are most problematic in relation to antibiotics, given that most antimicrobials used are antibiotics.

Among all the WHO regions, the risk of AMR infections is estimated to be highest in Southeast Asia (10). In Bangladesh, where this study is based, inappropriate practices in relation to the sale and use of antibiotics are widespread. For example, rural pharmacies appear to routinely sell antibiotics without requiring a prescription, despite this being illegal, and antibiotics are often illegally available from unregulated local shops selling medicines (11, 12). In rural areas public knowledge about antibiotics has been found lacking, while negative attitudes about the need to follow usage guidelines for antibiotics exist, and inappropriate practices reported (13). In rural communities where this study is based only half of adults even report being aware of antibiotics’ existence as a type of medicine (14), while among those adults who do claim to be aware of antibiotics and who report previously using them around 50% report having used them for a cough/cold (14), which are typically self-limiting, viral infections for which antibiotics are not appropriate (15). There is much less evidence on AMR and antimicrobial misuse in relation to small-scale, household-based livestock farming, but the little evidence that exists indicates that individuals frequently use antibiotics inappropriately as prophylactics and growth promoters for their animals, and that they access/purchase antibiotics for their animals when they are ill from informal drug shops (14, 16). Similarly, little research has looked at the environmental aspects of AMR from a community perspective in Bangladesh, but as with many LMICs unsafe practices related to infectious waste (e.g., feces) management are common (17), and studies have shown antibiotic resistant bacteria are frequently present in the environment in communities (18).

The WHO’s 2015 Global Action Plan on Antimicrobial Resistance includes as its first objective (of five) an objective to “Improve awareness and understanding of antimicrobial resistance through effective communication, education and training” (4). This explicitly covers communication and education of the public, as well as professionals. Similarly, Bangladesh’s National Action Plan: Antimicrobial Resistance Containment in Bangladesh 2021–2026 recommends that public communication and education about AMR is prioritized (this document is not publicly available at the time of writing, but members of our research team have access to it). However, it is critically important to go beyond simply raising public awareness of AMR and to actively engage with communities through community engagement approaches, to enable them to identify, develop and implement community-led and contextually-relevant, sustainable solutions (19, 20).

One proposed framework for community engagement emphasizes a high level of community participation, in which equitable partnerships are formed with community members and stakeholders, allowing them to identify, develop, and implement community-led sustainable solutions to issues affecting them and the wider global community, using existing or available resources (21). Based on these principles, an adaptable approach to community engagement was previously developed within rural communities in LMICs by the health research organization Malaria Consortium^1^, known as the community dialogue approach (CDA) (22–24). The goal of the CDA is to train “community-based volunteers … to facilitate regular community forums (referred to as community dialogues) where specific health issues affecting the community are explored, local solutions are identified, and participants collectively decide and plan how best to address the issue.” See *Intervention* below for more details.

Many studies have now looked at how community engagement approaches may address a range of health issues (e.g., (25, 26)). However, there has been relatively little focus on how effective they may be at addressing the drivers of AMR in community settings, with no such studies having been done in the Bangladeshi context that we are aware of. Previously, members of this research team, along with other colleagues, adapted the CDA to the rural Bangladeshi context to address AMR from a One Health perspective. This meant adapting the CDA to focus on community-level and individual-level behaviors and practices related to humans (e.g., sharing of antibiotics), animals (e.g., use of antibiotics to promote growth) and the environment (e.g., inappropriate disposal of antibiotics) that can cause/spread antimicrobial resistant microbes (27–29). This wider research team has also conducted extensive formative research on this intervention within this context using a range of mixed methods, including piloting and evaluating aspects of it on a small scale, establishing its acceptability and the feasibility of delivering it within this context (27–29).

Therefore, in this study we will evaluate this adapted CDA intervention, focusing on AMR from a One Health perspective, within rural Bangladeshi communities, on a large geographical scale, using a robust, pragmatic, randomized, controlled trial design, with associated process evaluation and costing studies. This will provide a comprehensive evaluation, under real-world conditions, of the effectiveness of the intervention and other key aspects of its functioning that are necessary to inform decisions around whether and how to scale the intervention up. Throughout the previous development of the intervention we have worked in an embedded way in collaboration with Bangladeshi government stakeholders (30), including policy makers and senior non-political members of relevant government departments, and we will continue to do so in this evaluation stage of the project. This embedded approach has been (and will continue to be) led by the Bangladeshi members of the research team, who lead/work for ARK Foundation (an independent health research organization)^2^ and the Faculty of Veterinary Medicine at Chattogram Veterinary and Animal Sciences University. This embedded approach will increase the likelihood that the resulting evidence will be used, and it will facilitate scale-up and sustainability of the intervention within the routine Bangladeshi health system, should the evidence support this decision.

This protocol was designed and reported in-line with the Standard Protocol Items: Recommendations for Interventional Trials (SPIRIT) guidelines (31). A completed SPIRIT checklist for this protocol is included (see Supplementary file 1).

### Objectives

Our first and main objective is to use the trial to evaluate whether our 12-month CDA intervention, focused on AMR from a One Health perspective, can improve levels of correct and appropriate knowledge about antibiotics, antibiotic use, and antibiotic resistance (ABR) from a One Health perspective, among adult community members within rural Bangladesh, as measured by our two primary outcomes. Our second objective is to use the trial to explore whether the CDA intervention may improve awareness of antibiotics, awareness of ABR, the presence of appropriate handwashing facilities in households, not sharing of antibiotics with family and friends, not using of antibiotics by individuals or their children for common, typically-viral, acute illnesses, not sharing household water sources with animals, and awareness of the risks of using antibiotic-containing feed for animals, as measured by our secondary outcomes. Our third objective is to carry out a process evaluation of the trial and intervention, using mixed methods and following the UK Medical Research Council’s guidance on developing and evaluating complex interventions, to help us interpret the results of the trial more comprehensively and effectively (32). Our fourth objective is to carry out a costing study of the intervention. Both objective three and four are primarily aimed at enhancing how we can use the results to inform considerations around future scale-up of the intervention.

### Trial design and key details

We will evaluate our intervention using a pragmatic, non-blinded, two-arm, parallel-group, cluster-randomized, controlled, superiority trial, with a repeated cross-sectional data collection design. Full details of the trial methods are below. In summary though, we will randomly allocate clusters, consisting of groups of rural communities (i.e., villages), to either an intervention or control group in a 1:1 ratio (with the randomization stratified by subdistrict). Within intervention clusters volunteer community members will be trained to facilitate a series of 11 distinct educational and discussion forums in each community (each repeated twice and delivered separately to female and male community members), over a period of 12 months, that aim to help their communities understand community-level and individual-level issues related to AMR from a One Health perspective and facilitate decision making around ways to tackle those issues. Control clusters will receive no trial related inputs other than the data collection activities required to collect the trial outcomes and other necessary trial data. We chose a cluster trial design as it is clearly not feasible to randomize delivery of the community education and discussion sessions at an individual level.

We will collect outcomes and other trial data from community members at baseline (pre-randomization) via a household survey. Following the intervention period we will then carry out an endline household survey of community members who were not included in the baseline survey. We will take this repeated cross-sectional approach (rather than a longitudinal approach) for two reasons. First, and most importantly, the baseline data collection will involve an extensive survey on reported knowledge and practices, and this data will be used to create our primary outcomes. We were therefore concerned that the baseline survey itself would have an impact on the effect of the intervention, at least in terms of the knowledge-related primary outcomes, by priming individuals about these issues. Therefore, to avoid contaminating the estimated treatment effect, particularly on the primary outcomes, we decided to ensure we would not follow-up the same individuals as we did at baseline. However, we recognize there may still be some impact of the baseline in this way through impacts diffusing at the community level. Second, we believe it would be challenging to follow-up the same individuals 12 months later and would likely result in quite a lot of missing data.

## Methods

### Study setting

This community-based study will be carried out within Cumilla district, south-east Bangladesh, about 100 km south-east of the capital Dhaka. According to the 2022 census (33) Cumilla has a population of 6,212,216 million. Among these individuals 53.3% are female and 46.7% male, 79.6% live in areas classed as rural and 20.4% live in areas classed as urban, and 76.5% of those aged 7 or older are classed as literate (75.7% of women and 77.5% of men). The district contains 17 subdistricts, known as upazilas, and 3,687 villages. The study will be based in five adjacent, primarily rural, subdistricts: Barura, Brahman Para, Burichang, Daudkandi, and Homna. These subdistricts were purposively selected for three reasons. First, based on the most recent census data (albeit from 2011) on literacy levels (among individuals aged 7+), these five subdistricts have literacy levels that are representative of the full range of subdistrict-level literacy levels found across all Cumilla’s subdistricts (see Supplementary Table S1 in Supplementary file 2). Second, the subdistricts were selected to be adjacent to each other to minimize costs and facilitate ease of access for the Bangladeshi research team (ARK Foundation (34)). Third, the Bangladeshi research team have prior experience of working within these subdistricts on community-based health research.

### Target population

The target population to whom we aim to make inferences are the adult (≥18 years) component of rural communities (i.e., villages) in Bangladesh.

### Cluster description and eligibility criteria

We pragmatically define our clusters as the adult (≥18 years) populations living within the catchment areas of community clinics (CCs). CCs are government-run, community health facilities that provide basic, primary healthcare services to rural communities across the whole of Bangladesh. CC population catchment areas typically cover several villages, usually containing around 6,000 individuals in total but with quite large variations. In our study subdistricts 76% of CC catchment areas covered 2 to 6 villages, while 24% covered 7 to 11 villages (median = 4). In a CC catchment area one village contains the CC building, which is typically a very basic and small structure. The services available at each CC are provided by one community healthcare provider. Community healthcare providers are classed as paramedics and are trained to provide basic healthcare, focusing on maternal and child healthcare, and when to refer patients up to higher levels of public healthcare. See *Community clinics: additional details* in for more details.

The CDA intervention will involve running a series of community-led health education and discussion forums separately within all the villages in each cluster. These forums will be delivered and facilitated by trained volunteer community members that we call facilitators. These facilitators will be supervised by either the community healthcare provider from the CC that covers the community in which they will deliver and facilitate the forums, or by their local health assistant or assistant health inspector (see Supplementary Table S4 in Supplementary file 2). Therefore, the catchment areas of CCs were chosen as a natural unit of clustering for the trial and intervention implementation.

To maximize the generalisability of the results to the target population all CC catchment areas within the study area will be eligible for inclusion in the trial.

### Participant description and eligibility criteria

To maximize generalisability to the target population the eligibility criteria for participants are also minimal, but they differ slightly between the baseline and endline surveys. For the baseline survey individuals will be eligible if they are aged ≥18 and have lived within the community that they are being sampled from for the past 12 months without having lived elsewhere for more than a month. However, for the endline survey individuals will be eligible if they meet the same criteria but they must also not have been a participant in the baseline survey, as explained in the *Trial design and key details* section.

#### Intervention description

**Figure 1:**
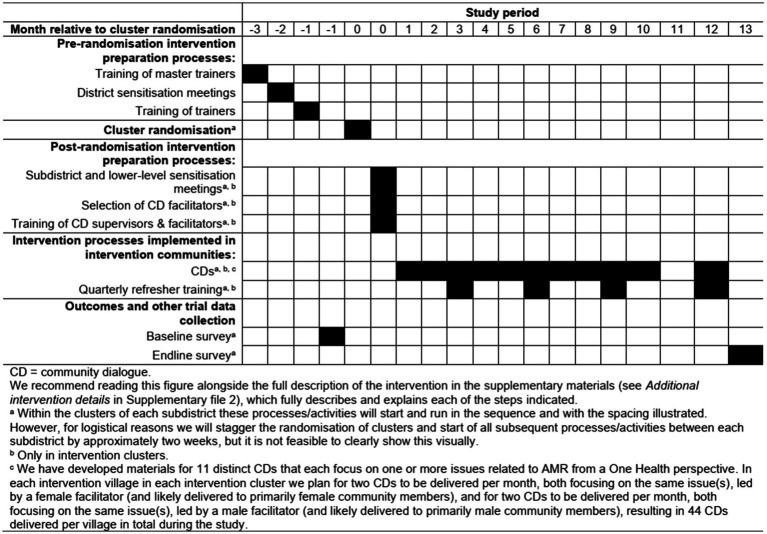
Trial planned timeline. CD community dialogue. We recommend reading this figure alongside the full description of the intervention in the Supplementary materials (see *Additional intervention details* in Supplementary file 2), which fully describes and explains each of the steps indicated. ^a^ Within the clusters of each subdistrict these processes/activities will start and run in the sequence and with the spacing illustrated. However, for logistical reasons we will stagger the randomization of clusters and start of all subsequent processes/activities between each subdistrict by approximately two weeks, but it is not feasible to clearly show this visually. ^b^ Only in intervention clusters. ^c^ We have developed materials for 11 distinct CDs that each focus on one or more issues related to AMR from a One Health perspective. In each intervention village in each intervention cluster we plan for two CDs to be delivered per month, both focusing on the same issue(s), led by a female facilitator (and likely delivered to primarily female community members), and for two CDs to be delivered per month, both focusing on the same issue(s), led by a male facilitator (and likely delivered to primarily male community members), resulting in 44 CDs delivered per village in total during the study.

Below we just present a summary of the key intervention details, while we report the full details of the intervention in the Supplementary materials (see *Additional intervention details* in Supplementary file 2), including the theory underpinning the CDA, how the CDA was embedded within the existing Bangladeshi health system structure, how the CDA will be run, and the planned focus of the different CDA forums that will be delivered. Also see the *Trial schedule* section below and Figure 1 for an overview of when the intervention processes and activities will occur within the study. We report our intervention guided by the Template for Intervention Description and Replication (TIDieR) guidelines for population health and policy interventions (35).

##### Community dialogue approach: development and adaptation

The CDA was originally developed by Malaria Consortium as an intervention for communities in LMICs. The overall idea was that “an iterative process of community dialogue and collective action work together to produce social change in a community that improves the health and welfare of all of its members.” (22) More precisely, in the CDA “community-based volunteers are train[ed] to facilitate regular community [forums] where specific health issues affecting the community are explored, local solutions are identified, and participants collectively decide and plan how best to address the issue. The approach is [intended to be] embedded within existing community and health structures” (22). This allows for social accountability (36), whereby community members can hold local (or possibly regional/national) health system stakeholders (e.g., public healthcare providers and managers and/or politicians) to account in relation to how the public health system is supporting communities to tackle the issues raised in the forums. It also allows local public healthcare providers to provide technical oversight of the forums. It is also designed to empower individuals by providing them with evidenced-based information on health issues that are relevant to them and their communities, and it provides a forum where they can ask questions about these issues, discuss them, and possible ways to address them, with other community members, and ultimately collectively agree on actions to try and address them. Over time, this is intended to “strengthen the relationship between communities and health providers” (22).

The CDA has been used successfully in other LMIC contexts to improve the use of health services and promote healthy behaviors for other health issues (37). As previously discussed, members of this research team and other colleagues previously adapted the CDA to the rural Bangladeshi context to address community-level issues related to AMR from a One Health perspective. This has included conducting extensive formative research on the adapted version of the intervention within this context using a range of mixed methods, including piloting and evaluating aspects of the intervention (but not in the same trial sites). This has demonstrated that this adapted CDA is acceptable to communities and local/regional/national stakeholders in this context, and that it is feasible to implement it within the health system and communities (27–29).

##### Pre-implementation work

Prior to implementing the intervention in the intervention communities we will carry out several stages of meetings with national-level, district-level, subdistrict-level and lower-level stakeholders, to sensitize them about the goals and details of the study and to obtain their support. We will then carry out several stages of training for certain stakeholders, including those community members who will ultimately deliver the CDA forums in their communities and the members of the health system who will support them (see below). The goals of this process are two-fold. First, a key goal is to embed the research within the Bangladeshi health system by involving governmental stakeholders from the design stage onwards, to increase the likelihood that the evidence from this study and prior work will be used by the health system to facilitate scale-up and sustainability of the intervention within the routine health system (30). Second, from a practical perspective the intervention requires key inputs and support from individuals in public health system roles to run successfully (e.g., community healthcare providers), which therefore requires governmental agreement and support.

##### Community dialogue approach: implementation

###### Community dialogues: overview, focus and frequency

Although the pre-implementation work described above will be critical and necessary for the intervention as a whole to function, the CDA will ultimately be operationalized and implemented in communities as a series of forums referred to as community dialogues (CDs), which are community-based and community-led forums for education, discussion, and collective decision making, with each CD focusing on one or more specific health issues related to different aspects of the overarching topic of AMR looked at from a One Health perspective. They will therefore cover knowledge, behaviors and practices related to tackling AMR within both the human, animal and environmental sectors. In this study the focus of the CDs is on understanding and addressing individual- and community-level issues related to AMR from a One Health perspective. The research team has developed content and key messages for 11 distinct CDs, each focusing on one or more distinct issues related to AMR from a One Health perspective (see *Community dialogue sessions key messages* in Supplementary file 2 for a list of the key messages that form the focus of each of the 11 distinct CDs).

As mentioned, the CDs will be facilitated by volunteer lay members from the community where they are delivered, known as facilitators. Based on local knowledge of the cultural context it is anticipated that female community members will be more likely to attend CDs facilitated by female community members and vice versa. Therefore, we aim to recruit one female and one male facilitator in every intervention village who will separately deliver CDs on the same focal issues in their village (although members of either sex will certainly be free to attend any CD if they wish). They will be asked to deliver the CDs in a public space, like a courtyard or school building. Within each intervention village we will ask each female and each male facilitator to both deliver two CDs per month, separated by approximately 2 weeks, with both CDs covering the same focal issue (i.e., repeating each of the 11 distinct CDs to maximize the reach of the CDA). The sequence in which each distinct pair of CDs are delivered will be the same for all facilitators. Therefore, allowing a month-long break for Ramadan, in each intervention village we expect each female and each male facilitator to deliver 22 CDs (covering 11 distinct focal issues) across a period of 12 months (see *Trial schedule* and Figure 1 for further details of the study’s planned timeline), giving a total of 44 CDs per village.

###### Community dialogues: delivery and facilitator support and monitoring

The facilitators will be trained to provide brief, evidence-based information in a lecture-style format about one or more focal health issue(s) to community members at the start of each CD, supported by their training, a facilitator guide and an accompanying pictorial flipbook (to visually illustrate the health issue(s) to participants). However, they will also be given the flexibility to tailor each CD to the specific needs and requirements of the community. As per their training, they will then facilitate discussions among community members about the health issue(s) covered and how they might address them within their community. The final goal is for each CD to conclude with the participants committing to one or more courses of action for them and their communities to undertake to help address the focal health issue(s) covered within their community, based on the collective discussions. The CDs are intended to be highly participatory and inclusive, with facilitators trained to encourage all participants to share their experiences, voice their concerns and contribute to decision making. Facilitators are also trained to encourage participants to spread information through word of mouth, set a positive example among family, friends and neighbors, and to hold each other to account for applying decisions reached during CDs.

Each facilitator will also be supervised by either the community healthcare provider from the CC covering the community in which they will deliver and facilitate the CDs, or by the Assistant Health Inspector (the supervisor of the community healthcare provider) of their subdistrict, or by a Health Assistant (basic healthcare provider and responsible for the Expanded Immunization Program at a local level) from their subdistrict. Facilitators will meet with their supervisor prior to their first CD to help plan, and then after every subsequent CD to discuss how the CD went, including any feedback from participants (which the facilitators will be trained to collect via a form), and to plan for the next CD.

We will also implement quarterly refresher training sessions for all facilitators and their supervisors, to refresh on the content of the CDs and address any issues faced by facilitators. The Bangladeshi research team will also recruit field officers who will attend facilitator supervisory meetings on an ad-hoc basis to ensure that the CDs are running effectively and to help solve any issues. These individuals will also help to maintain communications between facilitators and supervisors and the research team.

### Control description

Control group communities will not receive any inputs related to the trail other than the baseline and endline surveys, given that such community engagement activities are not the norm in this context and so this comparison reflects the existing situation. We are also not aware of any ongoing or planned community-based educational activities that relate to AMR or indeed any other topic in our trial communities.

### Outcomes

#### Outcome rationale and development

If we had no limitations, we would have selected one or more primary outcomes that allow us to understand if the intervention can reduce levels of antibiotic resistant infections in humans and animals, and levels of antibiotic resistant bacteria in the environment, in the communities where it is implemented. However, this was completely unfeasible given our resources and available timescales. The next proxy outcomes we would have liked to use would be based on individuals’ actual behaviors in relation to appropriate/safe antibiotic use (both for human and animal illnesses and antibiotic related practices relevant to environmental contamination). However, in most contexts, but particularly resource constrained contexts like rural Bangladesh, it is extremely challenging to collect such data as the only obvious ways would either be through direct observation, which would not be feasible logistically for this study, or having individuals log their usage behaviors, which we felt was also unfeasible given the high burden that would be placed on individuals (likely resulting in a very large proportion of missing data and/or biased outcomes).

Another possibility for less direct but related proxy outcomes would be to look at individuals’ reported practices in relation to antibiotic use, but the major problem with these types of outcomes are that, according to our data from our 2018 survey in this district, a large proportion of community members do not even know what an antibiotic is, and they are also likely to not be correctly informed when they are sold an antibiotic, particularly if purchasing medication from an informal drug seller, and so they would not necessarily even know if they purchased or used an antibiotic (14). Therefore, putting aside issues of measurement error, we did not want to focus just on reported practices as primary outcomes because these would only be collectable from individuals who knew what antibiotics were. Such antibiotic-aware individuals would clearly come from a subgroup of the general community that would not be representative of the whole community, and this would therefore not allow us to look at impacts across the whole community, which was a key part of our primary aim.

Therefore, we concluded that for our primary outcomes we would look at measures of correct and appropriate knowledge in relation to antibiotics and ABR in relation to human and animal health and the environment, as the CDs were explicitly designed to address and improve such knowledge. However, as no relevant measures of correct and appropriate knowledge exist for the topics and issues our unique set of CDs address, we had to create two bespoke primary outcomes measuring knowledge levels (see below). The questions used to create them were developed by members of the entire research team having specific subject matter expertise, with backgrounds in clinical practice, veterinary practice, public health, environmental-human health and water, sanitation and hygiene. The process of developing these outcomes involved first conducting two literature reviews.

First, we looked for any journal article conducting a survey in any LMIC on community members’ knowledge, attitudes and practices related to antibiotics within the last 10 years. Second, we collated the key messages for members of the public and farmers from recent guidance related to antibiotics, published by international and national organizations including the World Health Organization, World Organization for Animal Health (38), the European Union (39). We then looked at the content of the CDs and, alongside the findings of the two literature reviews, developed a set of questions that tested understanding of all the key messages we would want the full CD program to pass onto those attending CDs or hearing about them from family and friends. This set of questions was also refined by taking into consideration the local context in which the CDs would be run.

There then followed several rounds of iterative feedback and modification within the team. Lastly, we then pilot tested the draft questions with one female and one male from a similar rural community outside the study area. This pilot testing looked at how understandable the questions were and how acceptable and feasible the questionnaire was to the two participants. It also looked at the correctness of the skip patterns in the questionnaire. Based on the findings of the pilot testing we then amended and updated the questionnaire accordingly.

We developed our secondary outcomes in a similar manner, drawing on relevant subject matter expertise of team members, relevant literature and considering the local context, in an iterative process that was piloted before finalizing.

#### Primary outcome 1: percentage of correct/appropriate responses to 25 questions assessing knowledge about antibiotics, antibiotic resistance, and usage of antibiotics for human illness

Our first primary outcome measures individuals’ level of correct and appropriate knowledge about antibiotics (e.g., whether or not antibiotics can kill viruses, whether or not antibiotics can be delivered via pills, injections, creams etc), ABR (e.g., whether or not ABR can occur due to your body becoming “resistant” to antibiotics), and usage of antibiotics for human illness (e.g., whether it is appropriate and safe to share antibiotics with family/friends). We will ask survey participants a series of 25 factual, multiple-choice questions on these topics. Most of the questions will allow respondents to choose from a binary response (e.g., “yes” or “no,” or “appropriate” or “inappropriate”) but a few include more than two options, and all allow the respondent to choose “do not know.” Correct responses will receive a score of 1 and incorrect or “do not know” responses a 0 (see *Survey questionnaire* in Supplementary file 2 for a full list of the questions). We will then convert the overall score to a percentage of correct/appropriate responses for analysis. We are therefore implicitly treating all questions as equally important. This outcome will allow us to assess whether the CDs improve the level of correct and appropriate knowledge about antibiotics, ABR, and usage of antibiotics for human illness.

#### Primary outcome 2: Percentage of correct/appropriate responses to 20 questions assessing correct/appropriate knowledge about usage of antibiotics for animal health and antibiotic-related environmental considerations

Our second primary outcome will also measure individuals’ knowledge but will focus on correct and appropriate knowledge about usage of antibiotics for animal health (e.g., prophylaxis and growth promotion as well as disease) and antibiotic-related environmental considerations. For this outcome we will ask 20 multiple-choice questions, which will be scored in the same way as the previous knowledge outcome and again converted to a percentage for analysis (see *Survey questionnaire* in Supplementary file 2 for a full list of the questions). This outcome will allow us to assess whether the CDs improve the level of correct and appropriate knowledge about the usage of antibiotics for animal health and antibiotic-related environmental considerations (the other main aspects of a One Health perspective on antibiotics and ABR).

### Secondary outcomes valid for the entire sample

The following secondary outcomes are valid for the entire sample.

#### Antibiotic awareness

A binary indicator of basic reported awareness of the existence of antibiotics. Specifically, whether a participant responds “yes,” as opposed to “no/do not know,” to the question “Have you ever heard of a type of medicine known as an antibiotic or antibiotics?.” All our previous formative work in this setting indicated that there is no Bangla term for antibiotics and that the English term “antibiotic(s)” is most used, and hence was used for this question. In this setting survey data from 2018 indicated that only approximately half of the general rural adult population reported being aware of the existence of antibiotics as a type of medicine, based on a very similar question to the one we used (14). This outcome is to allow us to explore whether the CDs may improve the level of simple reported awareness of the existence of antibiotics, given an awareness of antibiotics is the most basic and fundamental starting point required to begin to understand issues related to antibiotic misuse and ABR.

#### Antibiotic/antimicrobial resistance awareness

A binary indicator of self-reported awareness of the existence of antibiotic/AMR. Specifically, whether a participant responds “yes,” as opposed to “no/do not know,” to the question “Have you heard of any of the terms ‘antibiotic resistance’, ‘antimicrobial resistance’ or ‘drug resistance’?” This outcome is to allow us to explore whether the CDs may improve simple reported awareness of the existence of ABR/AMR, given an awareness of ABR is the most basic and fundamental starting point required to begin to understand the problems associated with antibiotic misuse.

#### Presence of appropriate handwashing facilities in the household

A binary indicator of whether there was a handwashing facility in the participant’s household with water and either soap, alcohol gel, or detergent (either ash, mud or sand) or not. This was determined via visual inspection by the data collectors. This outcome is to allow us to explore whether the CDs may increase the relative frequency of the presence of appropriate handwashing facilities within households, which is one focal issue in the CDs.

### Secondary outcomes valid for subgroups of the sample

The following outcomes are only valid for a subgroup of the sample, or are restricted to only being collected from a subgroup of the sample, as described in each case.

#### Sharing of antibiotics with family/friends within the last 3 months

This outcome only applies to the subgroup of individuals in the sample who report that they are aware of antibiotics as a type of medicine. It is a binary indicator of whether the individual reports sharing any antibiotics with family/friends within the last 3 months. Specifically, whether the participant responded “yes” as opposed to “no” or “do not know” to the question “Within the last 3 months have you shared any antibiotics with any family, friends or neighbors?” This outcome is to allow us to explore whether the CDs may reduce the inappropriate sharing of antibiotics among family and friends, which is one focal issue in the CDs.

#### Personal use of antibiotics in the past 3 months for typically viral and self-limiting upper respiratory tract infections

This outcome only applies to the subgroup of individuals in the target population/sample who report being aware of what an antibiotic is, having suffered from either a “sore throat,” “chest cold” or a “common cold” in the past 3 months, and sought treatment for that illness. It is a binary indicator of whether that treatment included any antibiotics or not. These conditions should typically be viral and self-limiting, but it is known that antibiotics are frequently used to treat such conditions in this context, often due to individuals seeking care from informal drug sellers with poor/no training and a strong incentive to sell antibiotics. The CDs therefore emphasize seeking treatment from a qualified provider and only obtaining antibiotics using a prescription. Therefore, this outcome is to allow us to explore whether the CDs may decrease the relative frequency of inappropriate antibiotic use for these conditions among adults in this subpopulation.

#### Use of antibiotics in the past 3 months by any children (<15) of female community members for typically viral and self-limiting upper respiratory tract infections

This outcome only applies to the subgroup of female individuals, who report being aware of what an antibiotic is, who have one or more children <15 who have suffered from either a “sore throat,” “chest cold” or a “common cold” in the past 3 months, and the child was treated for that illness. The outcome is a binary indicator for each child (who has these characteristics) of whether the treatment included any antibiotics or not. These conditions should typically be viral and self-limiting, but it is known that antibiotics are frequently used to treat such conditions in this context, often due to individuals seeking care from informal drug sellers with poor/no training and a strong incentive to sell antibiotics. The CDs therefore emphasize seeking treatment from a qualified provider and only obtaining antibiotics using a prescription. Therefore, this outcome is to allow us to explore whether the CDs may decrease the relative frequency of inappropriate antibiotic use for these conditions among children in this subpopulation.

#### Sharing of water sources with animals

This outcome only applies to the subgroup of individuals in the sample who report that their household owns any animals. It is a binary indicator of whether animals can access and drink from the same source of water used by the participant’s household to drink and cook with. This was determined via visual inspection by the data collectors. This outcome is to allow us to explore whether the CDs may decease the relative frequency of such water sharing within households, which is one focal issue in the CDs.

#### Awareness of the benefits of not feeding healthy animals antibiotic-containing feed

This outcome is only collected from the subgroup of individuals in the sample who report being aware of what an antibiotic is and being aware of ABR. It is a binary indicator of whether the participant is aware that you can reduce the spread of antibiotic resistant infections by not feeding health animals feed containing antibiotics. Specifically, whether the participant responds “yes” as opposed to “no” or “do not know” to the question “Can you help to stop the spread of antibiotic resistant infections by not giving healthy animals feed containing antibiotics?” This outcome is to allow us to explore whether the CDs may improve simple reported awareness of the appropriate behavior in relation to this increasingly common but inappropriate animal-health related practice.

### Sample size

Due to logistical constraints around how many intervention clusters we believed we can implement in, we decided that we could have a maximum of 25 clusters per treatment group. As we plan to sample participants from two villages per cluster, and as we plan to sample an equal number of female and male participants per village, we also decided to sample an even number of participants per cluster. Following discussion among the trial team we decided that for both primary outcomes we would look at cluster-level treatment effects (40), which will mean aggregating the outcome to the cluster level by computing the mean of all survey participants’ percentage knowledge scores for each cluster (which we refer to as the cluster-level mean percentage knowledge score below). Based on discussion among the trial team we targeted being able to detect if the cluster-level mean percentage knowledge score for both outcomes was 15 percentage points or greater in the intervention group compared to the control group. We conservatively assumed an intracluster correlation coefficient of 0.3 for both primary outcomes, based on some related data from this setting in our previous survey study (14). We used the approach of Horzo to estimate plausible values for the overall variance for each primary outcome (41): 40 for the knowledge score measuring the level of correct and appropriate knowledge about antibiotics, ABR, and usage of antibiotics for human illness, and 25 for the knowledge score measuring the level of correct and appropriate knowledge about usage of antibiotics for animal health and antibiotic-related environmental considerations.

Based on these inputs and using the approach of (42) (based on formula 2 and assuming a cluster-level analysis), we then looked at a range of sample size scenarios. We adjusted the standard alpha level of 0.05 to 0.025, following a Bonferroni approach to account for the multiple testing of two primary outcomes. From these results we will aim to sample 44 participants per cluster (22 per village), so 1,100 participants across 25 clusters per treatment group and 2,200 participants across all 50 trial clusters, for the baseline and endline surveys. Conditional on these assumptions, when comparing between the treatment groups at endline we will have 90% power to detect a 14-percentage-point or greater difference between each group’s mean cluster-level percentage knowledge score regarding correct and appropriate knowledge about antibiotics, ABR, and usage of antibiotics for human illness, and 90% power to detect a 11-percentage-point or greater difference between each group’s mean cluster-level percentage knowledge score regarding correct and appropriate knowledge about usage of antibiotics for animal health and antibiotic-related environmental considerations.

### Sampling, recruitment and consent

#### Cluster sampling

Across all study subdistricts we have mapped the approximate geographical location of all clusters based on the location of their CCs. Based on our sample size calculation we have developed code, using the statistical software R (43), to carry out a stratified random sampling of 50 of those clusters while maximizing the minimum nearest-neighbor distances between all clusters to try and minimize the risk of contamination (e.g., individuals from control clusters traveling to intervention clusters to receive the CDs). Specifically, the code randomly sampled 10 clusters per subdistrict, with the restriction that none of the CCs selected were within the following minimum distances of any other CC within their respective subdistrict: 3 km for the subdistricts of Barura, Brahman Para and Burichang and 2.5 km for the subdistricts of Daudkandi and Homna. These minimum nearest-neighbor distances were maximized by iteratively running the sampling code and increasing the minimum-allowed nearest-neighbor distance for each subdistrict, in steps of 0.5 km, while ensuring that there were still ≥10 CCs available per subdistrict from which 10 were then randomly sampled per subdistrict. Table 1 gives the key numerical details of the cluster sampling process.

**Table 1:** Subdistrict-specific number of community clinics, maximum nearest-neighbor distance between community clinics used when sampling clusters, and number of remaining community clinics left once restricted by the maximum possible nearest-neighbor distance that still left ≥10 community clinics in the subdistrict.

Subdistrict	Total number of CCs	Minimum distance between CCs in restricted population	Number of CCs remaining for final selection of 10 clusters
Barura	29	3 km	15
Brahman Para	20	3 km	12
Burichang	24	3 km	13
Daudkandi	22	2.5 km	15
Homna	21	2.5 km	15

CCs, community clinics.

#### Cluster recruitment and consent

We will seek written or verbal consent for the participation of clusters from both the upazila chairman (the elected local government representative of the subdistrict) of each subdistrict and the union parishad chairmen (the elected local government representatives of each union within each subdistrict, where unions are the administrative level below a subdistrict in local government). We will seek this consent during subdistrict level trial sensitisation meetings, which we will hold in each subdistrict prior to sampling and randomizing clusters.

#### Participant sampling

We will collect all trial data via a baseline and an endline household survey within the selected clusters in each subdistrict (see *Trial schedule*). For the household survey we will use a pragmatic, multi-stage sampling approach to select participants, based on the older WHO EPI cluster sampling method informally referred to as the “spin the pen” method (9). Note that although the intervention will be run across all villages within each intervention cluster, we will only collect trial data from participants living in two (or potentially just one) village. Specifically, based on our sample size calculation, within each cluster we will aim to sample 22 participants from the village that the cluster CC is located within and a further 22 participants from the next nearest village in that same cluster (or if there is just one village in the cluster then all 44 participants will be selected from that village).

To sample participants from each selected village our data collection team will locate the approximate center of the village (using local knowledge as necessary) and then spin a pen on the ground to select a random direction. They will then walk a transect in that direction, sketch mapping all households immediately either side of that transect line until they reach the edge of the village. They will then number all the sketched households and, using a random number table, select a random household from those listed. They will then go to that household, locate the first available eligible individual, inform them about the study and seek their consent to participate in that survey round. If they agree they will then interview them. They will then locate the next nearest household, followed by the next nearest household in relation to that one, and so on, until they reach the fifth next nearest household from the one previously sampled.

They will then sample and interview the first available eligible individual at this household who is of the opposite sex to the first participant. This sex switching will then be repeated for each subsequent participant. This is to maintain an approximate 1:1 sex ratio. Although the sex ratio in the population being sampled should be approximately equal without this process we may end up with a moderate to large sex imbalance depending on the availability of females vs. males during the surveys. They will then repeat this household skipping selection process, while avoiding households they have previously sampled, until they have selected the required number of participants for that village.

If an eligible individual declines to participate then they will try and recruit another eligible individual in that household or move onto the next nearest household. This approach clearly does not mitigate against sampling bias due to differential participation, and this may affect the generalisability of the results, but it does maintain the target sample size and power. Where it is not possible to sample 22 or 44 individuals from separate households based on the household skip pattern, due to a lack of households, they will modify the skip pattern accordingly.

#### Participant recruitment and consent

Our data collectors will attempt to recruit the household members that they sample into the study, as part of either survey, after explaining the study to them and providing them with the trial information sheet (if they are literate). If they agree to participate, they will then seek consent to both participate in the trial via the survey and to allow us to make their anonymised data, collected during the survey, freely available for research purposes via a public research data repository.

### Randomization

The trial statistician (JPH) will randomize all sampled clusters to the intervention or control groups in an overall 1:1 ratio using a stratified and restricted approach (44). This will involve stratifying the randomization by randomizing clusters within each subdistrict separately in a temporally staggered, subdistrict-by-subdistrict sequence (see *Trial schedule*) for logistical reasons. Within each subdistrict we will simultaneously randomize the 10 sampled study clusters within that subdistrict in a 1:1 ratio (i.e., 5 per treatment group) using a restricted approach, which aims to balance (across treatment groups) the baseline cluster-level values of the first (primary outcome) percentage knowledge score regarding correct and appropriate knowledge about antibiotics, ABR, and usage of antibiotics for human illness. Given that we are randomizing relatively few clusters, this randomization approach will increase the chances of obtaining an effective balance between treatment groups in terms of both the expected endline primary outcomes under the counterfactual that the intervention is not applied, as well as other observed and unobserved characteristics that are strongly causally related to at least the primary outcomes (44).

Specifically, using the baseline survey data for each cluster we will first compute the baseline cluster-level mean percentage knowledge score from the percentage knowledge scores of all survey participants in that cluster. Following the approach described by (44), we will then develop R code to randomize the clusters in one subdistrict at a time subject to the following restriction. For any given subdistrict the code will first enumerate every possible way the 10 clusters in the subdistrict can be allocated between the treatment groups in a 1:1 ratio. Within each subdistrict there are ^10^C_5_ = 252 possible ways to allocate the 10 clusters in a 1:1 ratio. The code will then look at each of those allocations and for each allocation it will compute the mean of the cluster-level mean percentage knowledge scores for the clusters allocated to each treatment group, and the difference between those treatment group means. Lastly, it will randomly select one of the possible allocations where the difference between the treatment group means is ≤ ± 5 percentage points (i.e., where the means of the cluster-level mean percentage knowledge scores are similar between the treatment groups at baseline).

We will verify the validity of this restricted randomization process in two ways. First, for each subdistrict we will test whether the restricted randomization process has resulted in a sufficiently large set of possible allocations (out of the maximum 252 possible allocations) to allow a meaningfully random selection, by looking at the percentage of allocations among the 252 that satisfied the restriction criteria and were available for random selection (44). If this is less than 50% (126) we will relax the restriction criteria by allowing the chosen allocation to be selected from the set of allocations where the restriction criteria difference is ≤ ± 10 percentage points. If there are still less than 50% of the original allocations available to select from we will iteratively further increase the allowed restriction criteria difference by ±5 percentage point increments until 50% or more of the total possible allocations are available.

Second, for each subdistrict we will look at all the allocations that satisfy the restriction criteria and compute how often each pair of clusters are present in either the same or opposite treatment groups (44). If one or more pairs of clusters co-occur in the same treatment group ≥80% of the time across the available allocations then we will again relax the restriction criteria by allowing the selection to include allocations where the restriction criteria difference is ≤ ± 10 percentage points. Again, if this test still fails after relaxing the criteria as described we will iteratively further increase the allowed restriction criteria difference by ±5 percentage point increments until one or more pairs of clusters co-occur in the same treatment group <80% of the time.

### Masking

Due to the nature of the intervention, it is not possible to mask participants, those running the intervention or members of the research team to the allocation of clusters. However, the data collectors will explicitly not be informed about randomization outcomes for the villages they work in. It is perfectly plausible though that they will become aware of the treatment status of villages by talking to participants, who will clearly likely be aware if they are in an intervention village.

### Data collection, management and monitoring

#### Questionnaire

During the baseline and endline surveys we will collect all data via a digital questionnaire. A Microsoft Word version of the questionnaire is available in the Supplementary materials (see *Survey questionnaire* in Supplementary file 2). The questionnaire will record basic trial related data, such as the date and time, cluster and village identifier codes, and participant consent. It will also record a range of data on participants’ socio-demographic characteristics, such as their age, sex, education levels and animal ownership details. It will also record participants’ responses to the questions that will be used to generate the primary and secondary outcomes. Our endline survey questionnaire will also contain a series of questions about attendance and/or awareness of the CDs (both among participants and their family/friends etc.) to allow some of our planned subgroup analyses. The questionnaire was developed into an Android-based app using the commercial Survey CTO platform^3^, which in turn is based on the Open Data Kit^4^ platform. In the field the questionnaire will be completed by the data collectors via tablets running the Android operating system.

#### Data collection team

All data will be collected by two field teams, one created for the baseline survey and one created for the endline survey. Each team will consist of five or six enumerators (depending on how efficient the baseline team is), who will be chosen based on having experience of collecting data health survey data in these settings using tablets (we expect these individuals to be largely/entirely made-up of MSc students from Dhaka University studying health related degrees). The team will undergo a 5-day training program, delivered by members of the Bangladeshi research team and a member of the data collection app team. The training will cover the goals of the study, its design, the survey locations, the household and participant sampling methods, the questionnaire and data collection processes.

We will check their understanding and ability to collect data using a mock test on the survey questionnaire. This will allow us to address any weaknesses in their performance and any final issues identified with delivering the questionnaire. They will then practice sampling households and collecting data from a small number of community members in a similar field setting to the study sites, but in a site that will not form part of the study. There will then be a final day where the data collectors can discuss their learning from the practice field trip and solve any remaining issues.

#### Data quality

In the questionnaire app we will automate all required skips and build in a range of data formatting and sense checks to minimize errors when collecting data. The Bangladeshi research team, who are responsible for the running of the trial and data collection, will also make use of the Survey CTO platform’s ability to live stream audio of interviews to monitor a sample of initial interviews with each data collector. This will allow them to provide real-time and post-interview feedback to further improve the consistency with which interviews are conducted.

#### Data management

All data collected in the field will be transferred remotely via the internet to a secure server, from where it will be downloaded for processing. We will then carry out a standard series of data quality, accuracy and sense checks for each variable before creating a final dataset for analysis.

#### Trial monitoring and harms

The study sponsor is the University of Leeds (UK). We have created a trial steering group consisting of three senior researchers at the University of Leeds with relevant experience, who are within the School of Medicine but are not part of the project. Their remit is to provide advice aimed at the successful conduct of the trial, based on regular updates from the trail team. However, they will not look at any trial outcome data (although we will report monitoring data on the implementation of the interventions and on the progress of trial data collection). It was decided not to create a data monitoring committee because the design of the trial will involve a baseline and an endline cross-sectional survey. Therefore, there is no collection of trial outcome data throughout the intervention period, and so there is no opportunity to conduct interim analyses that may be used to stop the trial early. It was also not feasible to collect any data on potential harms.

### Trial schedule

Figure 1 illustrates the trial schedule. For logistical reasons, we will start by conducting the baseline survey in the clusters of one subdistrict before starting the baseline survey in the clusters of a second subdistrict approximately two-weeks later, and so on for the remaining subdistricts, such that all these activities will be collectively staggered by approximately 2 weeks between the subdistricts.

### Statistical analyses

Prior to database lock we will produce a statistical analysis plan. Therefore, below we just summarize the key details of the approaches we will use.

#### General principles

##### Participant and cluster characteristics

We will describe the cluster-level and individual-level distribution of relevant participant characteristics (e.g., age, sex, education level) across all cluster at baseline and endline, using standard descriptive statistics, to help inform judgments about the generalisability and transportability of the treatment effects. Using the same types of statistics, we will also describe the cluster-level and individual-level distribution of these characteristics across the clusters within each treatment group, to enable judgments about the balance of these characteristics between the treatment groups (and therefore the success of the randomization process).

##### Outcome treatment effects

When estimating treatment effects on our trial outcomes we will include all clusters and participants and analyze them according to their original cluster-randomized allocation, unless explained otherwise. This is to ensure that treatment effect estimates are pragmatic and reflect the likely effect of the intervention under real-world conditions, when communities and individuals may not participate in the intervention. It also preserves the benefits of the randomized design (45).

We will estimate all treatment effects based on a contrast between a suitable summary measure of the outcome in the intervention group compared to the control group at endline, and we will use baseline outcome and covariate data to create covariate-adjusted treatment effect estimates. We treat these covariate-adjusted results as our primary evidence about the causal effects of the intervention, as they typically provide increased precision and reduce the biasing impact of any imbalances between treatment groups in terms of the distributions of potential outcomes and participant/cluster characteristics that are causally related to the endline outcomes (46). We will also produce corresponding covariate-unadjusted estimates, where appropriate, and treat those as sensitivity analyses. As recommended by the *Consort 2010 statement: extension to cluster randomized trials* (47), for all binary outcomes we will compute treatment effects on both the difference and ratio scales (both adjusted and unadjusted for covariates).

When making judgments about the likely treatment effects in the target populations of interest we will focus on treatment effect point estimates and their 95% confidence intervals (48). However, for interested readers we will also compute and present the corresponding two-sided *p*-values for those treatment effects (computed based on the null hypothesis of no difference between treatment groups). We will also present a summary measure of each outcome within each treatment group at baseline and endline based on the summary measures contrasted for the treatment effect.

##### Missing data

Based on experience of conducting similar surveys in this content, we do not expect any missing data from participants who consent to being part of either survey. However, we do expect a small proportion of eligible individuals who we approach to participate in both surveys will refuse to do so. We will record and present data on the numbers of eligible participants who were approached to participate and the numbers who agreed, so that readers can judge whether there is likely to be any substantial impact of selection bias from this process on the results.

#### Primary outcomes

##### Main analyses

As our intervention targets communities and our interest is on the community level impact of the intervention, for our two primary outcomes we will estimate cluster-average treatment effects (40). Consequently, we will analyze our two knowledge score primary outcomes using cluster-level analysis methods that are suitable for stratified, cluster-randomized, controlled trials (44, 49). This approach allows us to estimate covariate-adjusted and covariate-unadjusted cluster-average treatment effects that account for the clustered structure of the study design and data.

For our covariate-adjusted analyses for each primary outcome we will use a two-stage method that first involves creating an ordinary least-squares linear regression model with the survey participants’ percentage knowledge scores as the outcome variable and a range of individual-level and cluster-level variables as adjustment covariates (see below), including a variable identifying the strata (subdistricts), but excluding the treatment group variable. Using the model, we would then compute model-predicted outcome values for each survey participant (i.e., their predicted knowledge score percentage), based on their observed covariate values. We would then take the mean of the individual-level predicted outcome values within each cluster to create cluster-level model-predicted outcomes values. We would also take the mean of participants’ observed percentage knowledge scores within each cluster to create cluster-level observed outcome values. We would then subtract the cluster-level observed outcome values from the cluster-level model-predicted outcome values to create cluster-level difference residuals (44). In the second stage we would then estimate the (covariate-adjusted) cluster-average treatment effect via a second ordinary least-squares linear regression but with the cluster-level difference residuals as the outcome and one covariate for the clusters’ treatment group, based on the regression coefficient for the treatment group covariate along with its t-based 95% confidence intervals and two-sided *p*-value (correcting the degrees of freedom for the stratified approach) (49).

In these analyses we will adjust for the following covariates: sex (female/male), age (years) and age^2^, education level (none, less than primary, primary, less than secondary, secondary or less than higher secondary, higher secondary, graduate/post-graduate), employment status and main role in the last 30 days (no paid/in-kind work, nonprofessional/manual, personal business, professional), ownership of any animals by the household for food/economic purposes (yes/no), number of individuals living in the household, and the cluster-level baseline mean of the primary outcome being analyzed. We chose these variables as being plausibly prognostic of our outcomes.

We will also produce covariate-unadjusted cluster-average treatment effect estimates for the two primary outcomes using the same approach but excluding all covariates in the first regression model other than one identifying the strata.

We will adjust both the *p*-values and the confidence intervals for the multiple testing of the two primary outcomes via the Bonferroni approach separately for both the covariate-adjusted and covariate-unadjusted analyses.

##### Effect modification due to baseline characteristics

We will also explore whether the treatment effects for the two primary outcomes vary by two key individual-level baseline characteristics: sex (female/male) and reported education level (secondary or higher vs. less than secondary). For each primary outcome and each potential effect modifier we will first estimate the covariate-adjusted and covariate-unadjusted cluster-average treatment effect for each of the two subgroups using the cluster-level analysis methods described above (*Main analyses*).

Following the approach of (44) for each potential effect modifier we will then estimate covariate-adjusted and covariate-unadjusted cluster-level measures of treatment effect modification. For the covariate-adjusted analysis this will involve computing the difference between the cluster-average treatment effect for the two subgroups as an estimate of how the cluster-average treatment effect differs between those subgroups (i.e., the cluster-level measure of treatment effect modification). Similar to the cluster-level covariate-adjusted analysis approach described in the *Main analyses* section above, this will involve using an ordinary least-squares linear regression model to adjust for covariates (including the strata) other than the treatment group but also excluding the effect modifier. Using the model, we will then extract the covariate-adjusted residuals for each cluster separately for participants in each effect modifier subgroup and summarize them separately via the mean. We will then compute the difference between these cluster-level subgroup-specific means and use them as the outcome variable in a second ordinary least-squares linear regression model with one covariate for treatment group, with the coefficient for treatment group now giving an estimate of the difference between the cluster-average treatment effect estimates for the two subgroups (i.e., the cluster-level measure of treatment effect modification), along with an associated 95% confidence intervals and *p*-value (with the same adjustment for the stratified approach) (44, 49).

We will treat these effect modification results as exploratory and therefore not adjust them for the multiple testing of the primary outcomes.

##### Effect of engagement with the community dialogue approach intervention

As we do not expect all adult community members to engage with the CDA intervention we will also estimate the individual-level causal effect on the two primary outcomes of (1) having attended one or more CDs (yes/no) and (2) the number of CDs attended (i.e., the linear relationship between the number of CDs attended and the expected mean of the primary outcome). As these treatment effects are non-randomly assigned we will use an instrumental variable approach to try and account for likely confounding, which will estimate these two causal effects among the population of individuals who would be predicted to always comply with the intervention if it was available (i.e., attend the CDs if they were available in their communities) (50). These types of treatment effects are therefore often referred to as complier average causal effects or local average treatment effects (50). Specifically, we will use the two-stage residual inclusion instrumental variable approach with cluster treatment allocation as the instrument, as opposed to the more common two-stage least squares approach, which is more suitable for modeling binary treatments and non-continuous outcomes (51).

To estimate the causal effect on both primary outcomes of attending one or more CDs the two-stage residual inclusion process will involve first fitting a generalized linear model with Bernoulli errors and a logit link to an outcome indicator of whether a survey participant reports having attended one or more CDs, with covariates for cluster treatment allocation, subdistrict, sex (female/male), age (years) and age^2^, education level (none, less than primary, primary, less than secondary, secondary or less than higher secondary, higher secondary, graduate/post-graduate), employment status and main role in the last 30 days (no paid/in-kind work, nonprofessional/manual, personal business, professional), ownership of any animals by the household for food/economic purposes (yes/no), number of individuals living in the household, and the cluster-level baseline mean of the relevant primary outcome. We will then use the model to compute every participants’ model-predicted outcome value based on their observed covariate values, and then subtract those predicted outcomes from a numeric indicator of whether the participant attended one or more CDs (coded as 1) or not (coded as 0) to create residuals for each participant.

We will then fit a second-stage generalized linear model with Poisson errors and a log link to the primary outcome of interest. However, instead of the percentage knowledge scores as outcome values we will analyze the raw count of correct/appropriate answers, as appropriate for the Poisson distribution. The model will contain covariates for whether the participant reported attending one or more CDs (yes/no), the residual computed in the first-stage model, and all the other covariates included in the first-stage model.

Using this model, we will then use marginal effects methods to estimate the treatment effect by computing each participants’ model-predicted count of correct/appropriate answers based on their observed covariate values except for attending one/more CDs, which will be first set to yes for all participants (and predictions saved) and then no for all participants (and predictions saved). We will then convert these predicted counts of correct/appropriate answers to predicted percentages of correct/appropriate answers by dividing by the relevant number of questions asked for that knowledge score and multiplying by 100. Lastly, we will take the mean of each set of predictions and take their difference. This will estimate the causal effect of attending one/more CDs as the mean difference in the relevant percentage knowledge score for those attending one/more CDs compared to those attending no CDs, among those who would be predicted to always attend CDs if they were available in their community.

As a sensitivity analysis we will also repeat these analyses but at the second stage use generalized linear models with Gaussian errors and identity links instead, with the relevant percentage knowledge scores as the outcome, to compare with the results based on the Poisson model.

For statistical inference we will estimate 95% confidence intervals for all these treatment effects via a stratified, clustered bootstrap approach (52), to account for the clustered structure of data. This will involve resampling clusters within subdistricts while maintaining a 1:1 treatment allocation of the clusters within each bootstrapped subdistrict dataset, and using a modification of the bias-corrected accelerated method to compute the 95% confidence intervals from the bootstrapped data (53) (based on 5,000 resamples).

For an instrumental variable analysis to produce an unbiased estimate of the treatment effect of interest there are four key assumptions that must be met (54). Although there are many other possible sources of bias, and meeting these assumptions does not guarantee an unbiased estimate, we believe the analysis will not at least not violate these assumptions in any substantial way. (1) The instrument must be conditionally randomized with respect to the outcomes of interest and the treatments of interest. As we will be using the random allocation of clusters to the assigned treatment group as the instrument this assumption will be satisfied. (2) The instrument must effect the treatments of interest. Unless there are no survey participants in the intervention group communities who engage with the intervention this assumption will be satisfied. (3) The instrument must have no direct effect on the outcomes of interest and only affect them indirectly via the treatments of interest (commonly known as the exclusion restriction). As we are using the random allocation of clusters to treatment groups as the instrument this random allocation itself clearly has no causal effect on the outcomes of interest (only the treatment implemented, which may or may not be received by a participant), and so this assumption will be satisfied. (4) There are no survey participants who would attend CDs if they were available in their communities and if they were not encouraged to attend, but who would, conversely, not attend any CDs if they were available in their communities and if they were encouraged to attend (the community volunteers running the CDs will be encouraged to encourage members of their community to attend CDs). This is more speculative but, given the unusual behavior required, if such individuals do exist we believe they will be very infrequent.

#### Secondary outcomes

##### General considerations

We will not adjust any of these results for multiple testing and treat them as exploratory. We do not plan to compute effect modification results for any secondary outcomes but we may do so as a strictly exploratory process for any secondary outcomes if we find clear evidence of a treatment effect.

##### Whole sample secondary outcomes

Both of our secondary outcomes that apply to the whole sample (1. antibiotic awareness and 2. the presence of appropriate handwashing facilities in the household) are binary. For these secondary outcomes we will also estimate covariate-adjusted and covariate-unadjusted cluster-average treatment effects using similar two-stage cluster-level analysis methods as described above (see *Main analyses*) but adapted for binary outcomes to produce treatment effect measures on the risk difference scale (44, 49). For both sets of results the only difference in the approach is that in the first stage we will use a generalized linear model with Bernoulli errors and a logit link to compute the cluster-level model-predicted outcome values by taking the mean of the individual-level model-predicted outcome probabilities within each cluster, but the rest of the method remains the same.

These risk difference scale treatment effects will be our primary focus but, as recommended by the *Consort 2010 statement: extension to cluster randomized trials* (47), we will also compute both covariate-adjusted and covariate-unadjusted cluster-average treatment effects for these binary outcomes on the ratio-scale (as supporting auxiliary results). We will again use the same broad two-stage approach for both sets of results but slightly further modified.

Specifically, in the first stage we will again use a generalized linear model with Bernoulli errors and a logit link to compute the cluster-level model-predicted outcome values, but instead of creating cluster-level difference residuals we will create cluster-level ratio residuals by dividing the cluster-level observed mean outcome values by the cluster-level model-predicted outcome values. Then in the second stage we will again use an ordinary least-squares linear regression but with the natural logarithm of the cluster-level ratio residuals as the outcome and a covariate for treatment group. Lastly, by transforming the regression coefficient for the treatment group covariate along with its t-based 95% confidence intervals via the exponential function we then obtain an estimate of the treatment effect on the risk ratio scale (technically as a ratio of the geometric mean of the outcome in each treatment group) (44, 49).

##### Subgroup-specific secondary outcomes

Our remaining secondary outcomes are only valid for subgroups of the sample, such as those individuals who report being aware of what an antibiotic is. For all these outcomes the sample can be divided into individuals within the subgroup where the outcome is valid and individuals not in that subgroup. As these subgroups may be at least partly determined post-randomization by the intervention (e.g., the intervention may well affect whether an individual in the intervention group is aware of what an antibiotic is) a standard analysis of the treatment effect, by simply subsetting the data, would be prone to post-randomization selection bias (55). For these secondary outcomes we will therefore use a slightly-modified, regression-based approach that aims to adjust for this potential selection bias when estimating cluster-level treatment effects. In the literature these are known as survivor average causal effects, as these types of methods were originally developed to address selection bias in the context of survival/death creating subgroups of individuals where the outcome is valid or not (e.g., you cannot measure health outcomes if the patient has died) (56).

Our approach aims to estimate the cluster-average causal effect of treatment among the subgroup of individuals who would be part of that subgroup irrespective of any effect of the intervention (e.g., among individuals who would be aware of what antibiotics are irrespective of any effect of the intervention on their awareness of what antibiotics are). As with our whole-sample secondary outcomes, we will compute these treatment effects on both the absolute difference and ratio scales as the difference/ratio in the cluster-level percentage occurrence of the relevant outcome event between treatment groups, among the subgroup of individuals for whom the outcome is valid.

See (56) for full details of the original method. In summary though, we will first fit a generalized linear model with Bernoulli errors and a logit link to the binary indicator of whether an individual is in the subgroup where the ultimate outcome of interest is valid not (e.g., whether they report being aware of what an antibiotic is or not, with the ultimate outcome of interest being whether they report being aware of what ABR is). The model will contain covariates for cluster treatment allocation, subdistrict, sex (female/male), age (years) and age^2^, education level (none, less than primary, primary, less than secondary, secondary or less than higher secondary, higher secondary, graduate/post-graduate), employment status and main role in the last 30 days (no paid/in-kind work, nonprofessional/manual, personal business, professional), ownership of any animals by the household for food/economic purposes (yes/no), number of individuals living in the household, and the cluster-level baseline mean of the relevant secondary outcome. We will then use the model to compute outcome probabilities for every participant and subtract these from 1 to create an adjustment factor that will be used in the next stage to adjust for any selection bias due to the subgroup being determined post-randomization.

We will then subset the data to only include those participants who are in the subgroup for which the ultimate outcome is valid, and then fit a generalized linear model with Bernoulli errors and a logit link to the relevant ultimate secondary outcome of interest (e.g., reported awareness of what ABR is). This model will include covariates for the selection bias adjustment factor and all the covariates included in the first stage model. Unlike in (56) this model will also be weighted by the inverse of the cluster sample sizes to produce cluster-average causal effect estimates (40). Then, as described above (*Effect of engagement with the community dialogue approach intervention*), we will use marginal effects methods to compute survivor average causal effect estimates on the difference and ratio scales. Lastly, we will compute 95% confidence intervals for all these treatment effect estimates based on the same clustered bootstrap approach described above (*Effect of engagement with the community dialogue approach intervention*).

#### Interim analyses

There are no interim analyses planned.

## Process evaluation

We will conduct an embedded process evaluation based on MRC guidance (57). We will analyze the community, cultural, human health, animal health and environmental health system contexts in which the CDA is implemented. We will describe implementation processes in terms of fidelity, dose, adaptation and reach, and examine potential causal mechanisms of impact, with a focus on participant responses to and interactions with the intervention. We will use a range of qualitative and quantitative methods to address these goals. This will include document review (e.g., monitoring records) and observations of training sessions and CDs. We will also run focus group discussions and conduct semi-structured interviews with key stakeholders and community members, based on the purposive selection of participants.

## Economic evaluation

We will conduct an economic evaluation that will seek to do two things. First, we will undertake a costing of the CDA including the development of tools, training of master trainers and facilities and carrying out the dialogue process itself. The costing will impute the costs of unfunded inputs such as volunteer time to provide a realistic idea of the overall cost to society of the intervention. The costs will be used to understand how much the intervention might cost if used at scale across the country to change antimicrobial behavior. The second part of the component will be to compute cost effectiveness ratios that can be used to understand the relative cost-effectiveness of the CDA intervention compared with other ways of changing antimicrobial behavior. The information from the economics component may later be used to model the cost-effectiveness of the interventions that are developed from the dialogue process to change the use of antimicrobials, and ultimately impact human and animal health and the wider economy.

## Dissemination plans

We will update the trial registry details with the key outcome results from the trial. We will primarily disseminate the results to the scientific community via peer-reviewed publications (including at least one for the trial outcome results, one for the process evaluation results and one for the economic evaluation results). For all publications all authors will need to have met the International Journal of Medical Committee Editors authorship guidelines. We will also seek to disseminate the various results via scientific conferences and on smaller scales via seminars.

## Discussion

Community engagement approaches have successfully addressed other health issues in low resource settings (26), but there is limited evidence on using community engagement approaches to address ABR in low resource contexts, particularly in Bangladesh. In this study we will operationalize a One Health approach to improving antimicrobial resistance in a low-resource rural setting. The community dialogue approach we will take was deliberately adapted to address human, animal and environmental practices that drive AMR, and the evaluation will therefore provide evidence on how integrated community-based interventions can contribute to the wider One Health agenda (27–29). More specifically, the intervention explicitly covers knowledge across human, livestock and environmental domains. This includes addressing inappropriate antibiotic use for viral illnesses in humans, the use of antibiotics as prophylactics or growth promoters in animals, and community-level practices such as unsafe disposal of medicines and waste. By improving knowledge about these interconnected drivers simultaneously, the intervention moves beyond siloed approaches that have historically characterized AMR interventions (26).

The trial will also empower communities to identify locally relevant One Health risks and solutions through participatory dialogues and discussion facilitated by community members (37). This community-led process encourages collective action, such as separating animal and household water sources, following qualified health providers’ advice, and implementing safer waste management, which represents a practical and scalable model for community engagement in One Health. By embedding the intervention within existing community clinic catchment areas and linking facilitators to government health staff, the study also strengthens connections between communities and the health system across multiple sectors. In doing so, it demonstrates how One Health principles can be operationalized within routine service delivery structures in Bangladesh. Finally, the study will generate robust evidence on effectiveness, cost and feasibility of a One Health intervention in an LMIC context. While global and national AMR strategies emphasize the importance of One Health, there is limited empirical evidence on how this can be implemented at community level (26). Therefore, the findings from this trial will have relevance both for scaling within Bangladesh and for informing global debates on how to operationalize One Health approaches to AMR in comparable settings.

The study should have several key strengths. First, the use of a cluster-randomized controlled design, which will support robust inferences about the causal effects of the intervention (44). Second, stakeholders from the Bangladeshi health system have been closely involved (embedded) in this research from the very start of the wider project, which should help to ensure its feasibility and facilitate scale-up (30). Third, the relatively large number of communities that will be involved and the geographical area of the study should facilitate robust generalizations beyond the target population to the general rural population of Bangladesh. Fourth, the pragmatic design of the intervention and study should facilitate real-world inferences about any likely effects if the intervention is to be scaled-up in practice under real-world conditions (30).

The study also has several potential limitations. First, possibly the most important design limitation will be the primary reliance on knowledge-based outcomes, rather than objective practice−/behavior-based outcomes, which we could not feasibly measure due to the various challenges discussed above. Inevitably, this means that any impacts of the intervention will not be on the ultimate outcomes of interest, which will weaken our ability to fully justify scaling the intervention, should it prove effective. Second, we decided to rely on cross-sectional data rather than longitudinal data, which means our causal effects must rely on average group-level changes rather than average individual-level changes. However, as explained previously we believe this choice was preferable to risking contaminating our treatment effect estimates with any effect of the survey itself. Third, probably the most important potential unintended side effect would be if the intervention caused people to avoid taking antibiotics when they or their family members needed them, or if it caused them to avoid giving antibiotics to their animals if they needed them. However, we explicitly designed the intervention materials with this in mind. For example, as part of the key messages delivered during the CDs it will be emphasized that antibiotics are extremely important, potentially life-saving drugs, if used properly, and that individuals who are ill should always try to seek the care of a qualified healthcare provider and follow their advice.

## Trial status

We collected our baseline data between 21/9/2022–24/11/2022 and expect to complete our endline data collection by 31/5/2024.

## Protocol amendments

We will describe any important protocol amendments in our trial results paper, as recommended by the CONSORT guidelines (47).

## Footnotes

1
https://www.malariaconsortium.org/

2
https://arkfoundationbd.org/

3
https://www.surveycto.com

4
https://opendatakit.org

## Author’s note

The corresponding author had full access to all the data in the study and had final responsibility for the decision to submit for publication.

## Supplementary material

SUPPLEMENTARY TABLE 1 SPIRIT trial protocol checklist.
