# Supplementary material for: Engaging rural communities in Bangladesh to address antimicrobial resistance via the community dialogue approach: a protocol for a cluster-randomized controlled trial
Source: Front Public Health. 2025 Dec 17;13:1432635. doi: 10.3389/fpubh.2025.1432635 (PMC12753979; doi:10.3389/fpubh.2025.1432635)
Supplement: SUPPLEMENTARY TABLE 2 — Additional information. [file Table_2.DOCX]

Engaging rural communities in Bangladesh to address antimicrobial resistance via the Community Dialogue Approach: a protocol for a cluster-randomised controlled trial

Additional information

**Contents**

[1. Literacy levels in the subdistricts of Cumilla district 3](#_Toc163059127)

[2. Community clinics: additional details 4](#_Toc163059128)

[3. Additional intervention details 5](#_Toc163059129)

[3.1. Community Dialogue Approach: theory 5](#_Toc163059130)

[3.2. Community Dialogue Approach: practice 5](#_Toc163059131)

[3.3. Pre-implementation work 6](#_Toc163059132)

[3.3.1. National-level governmental stakeholder sensitisation and collaboration 6](#_Toc163059133)

[3.3.2. Training of master trainers 7](#_Toc163059134)

[3.3.3. District-level sensitisation meeting 8](#_Toc163059135)

[3.3.4. Training of trainers 9](#_Toc163059136)

[3.3.5. Sensitisation of subdistrict-level and lower-level stakeholders 9](#_Toc163059137)

[3.3.6. Selection of supervisors and facilitators 13](#_Toc163059138)

[3.3.7. Training of facilitators and supervisors 13](#_Toc163059139)

[3.4. Implementation and delivery of Community Dialogues 14](#_Toc163059140)

[3.4.1. Community Dialogue delivery structure 14](#_Toc163059141)

[3.4.2. Facilitator supervision and Community Dialogue monitoring and evaluation 14](#_Toc163059142)

[4. Survey questionnaire 16](#_Toc163059143)

[4. Community dialogue sessions key messages 46](#_Toc163059144)

[References 50](#_Toc163059145)

# 1. Literacy levels in the subdistricts of Cumilla district

| **Table S1. Subdistrict-level literacy levels among individuals aged 7+ within Cumilla district** | | |
| --- | --- | --- |
| **Subdistrict** | **Population** | **Literacy among individuals aged 7+** |
| Homna* | 206,386 | 39.7 |
| Titas | 184,617 | 43 |
| Meghna | 112,453 | 44.6 |
| Muradnagar | 523,556 | 48.8 |
| Daudkandi* | 349,910 | 50.7 |
| Chandina | 350,273 | 51 |
| Nangalkot | 373,987 | 51.2 |
| Laksam | 253,650 | 51.5 |
| Barura* | 405,118 | 52.1 |
| Debidwar | 431,352 | 52.8 |
| Brahman Para* | 204,691 | 54.7 |
| Comilla Sadar Dakshin | 258,278 | 55.1 |
| Manoharganj | 244,943 | 55.9 |
| Chauddagram | 443,648 | 56.9 |
| Burichang* | 301,825 | 57 |
| Comilla Adarsha Sadar | 532,419 | 65.7 |
| *Study subdistricts. | | |

# 2. Community clinics: additional details

A key part of the government-delivered training programme for community health care providers of community clinics (CCs) focuses on prescribing a small range of key antibiotics (e.g. amoxicillin, doxycycline, cotrimoxazole and metronidazole) correctly (e.g. screening out uncomplicated upper respiratory tract infections, and identifying when patients have conditions requiring the use of the antibiotic).

Based on the national policy governing CCs, the aim is for each CC to be managed by two sets of groups made up of representatives from the community: one “community group” and up to three “community support groups”, with each group having up to 17 members. As specified by the policy, the members of these groups are supposed to represent a broad spectrum of the local population, including males and females, and individuals of different ages, and from different socio-economic groups and professions. The key responsibilities of the community groups are to manage CC organisational issues including opening and closing times, medicine supply management, infrastructural issues such as the electricity supply, and respond to any community concerns. While the key responsibilities of the community support groups are to inform the community, particularly mothers, about the services that the CC can provide, to help the community access the CC in emergency situations, run community engagement activities, and to raise funds from the community to support the CC. However, in practice not all CCs have fully functional community groups and/or community support groups.

# 3. Additional intervention details

The intervention description below is guided by the Template for Intervention Description and Replication (TIDieR) guidelines for population health and policy interventions (1). Below we briefly describe the theory behind the CDA as a general intervention followed by how we developed this specific version of the CDA and the intervention we will implement and evaluate in this trial.

## 3.1. Community Dialogue Approach: theory

The Community Dialogue Approach (CDA) was adapted from the Integrated Model of Communication for Social Change by Malaria Consortium to help communities in low- and middle-income countries address issues affecting their communities (2, 3). In this model it is assumed that a stimulus is needed to trigger dialogue among members of a community about the issues concerning the community. Here, dialogue is understood to be a dynamic and iterative process, which ideally results in collective decision making about how to resolve the issues of concern. The dialogue is ultimately assumed to cause social change by increasing the self-efficacy of individuals and the community, by strengthening community ownership, and by shaping social norms. The CDA is therefore a very general intervention that can be used to address all kinds of issues communities face, including but not restricted to health issues. However, in this study the focus was on addressing community-level issues related to antimicrobial resistance (AMR) and the potential problems it can cause for communities, and the CDA was therefore adapted with this focus in mind (4).

## 3.2. Community Dialogue Approach: practice

In the CDA “community-based volunteers are train[ed] to facilitate regular community meetings where specific health issues affecting the community are explored, local solutions are identified, and participants collectively decide and plan how best to address the issue.” (5) These meetings are known as community dialogues (CDs), which are community-based and community-led forums for education, discussion (hence why they are referred to as community dialogues), and decision making, with each CD focusing on one or specific health issues. In this study all the specific health issues that will be covered are part of the overarching issue of AMR from a One Health perspective. The CDs are facilitated by volunteer lay members from the community where they are delivered, known as CD facilitators (facilitators from hereon).

The facilitators are trained to provide brief, evidence-based information in a lecture-style format about one or more focal health issue(s) to community members at the start of each CD, supported by their training, a facilitator guide and an accompanying pictorial flipbook (to visually illustrate the health issue(s) to participants). However, they are also given the flexibility to tailor each CD to the specific needs and requirements of the community. They are trained to then facilitate discussions among community members about the health issue(s) covered and how they might address them within their community. The final goal is for each CD session to conclude with the participants committing to one or more courses of action for them and their communities to undertake to help address the focal health issue(s) covered within their community, based on the collective discussions. The CDs are intended to be highly participatory and inclusive to give all participants the opportunity to share experiences, voice concerns and contribute to decision making. Facilitators are also trained to encourage participants to spread information through word of mouth, set a positive example among family, friends and neighbours, and to hold each other to account for applying decisions reached during CD sessions.

In the CDA the triggering stimulus for change is therefore assumed to be both external (provision of training and tools for the facilitators) and internal (selection of volunteers to facilitate the CDs, and volunteers mobilising participants to attend CDs) to the community. The community engagement process is also “… embedded within existing community and health structures allowing for social accountability and technical oversight. It is designed to empower individuals, and over time strengthen the relationship between communities and health service providers.” (5)

## 3.3. Pre-implementation work

A key goal of this study is to embed the research within the Bangladeshi health system by involving governmental stakeholders from the design stage onwards. This is for two main reasons. First, key aspects of the intervention (both pre-implementation and during implementation) will be delivered by individuals in public sector roles (e.g. community health care providers). Therefore, we require their support and inputs for the intervention to run successfully, which therefore requires governmental support (i.e. the agreement and support of the relevant leaders, in those sectors and at the relevant levels, that those individuals can and should undertake these activities within their job role). Second, longer-term this embedded approach should facilitate usage of the evidence generated by the research by the government, and therefore the eventual scale-up and sustainability of the intervention within the routine health system (6).

Specifically, both the professional and community members involved in running the CDs need to be recruited and trained by relevant experts in relevant public sectors. Therefore, the research team will initially train a set of master trainers who will then train a larger set of trainers who will finally train the voluntary facilitators (who will be recruited from their communities) and their supervisors (who will be recruited from the public health sector). Below we describe the sensitisation and training processes necessary for this to occur in its planned chronological order.

### 3.3.1. National-level governmental stakeholder sensitisation and collaboration

Initially, the Bangladeshi research team held a series of meetings with national-level governmental stakeholders in Dhaka. The goal of these meetings was to explain the rationale and importance of the project and to seek government support. The meetings primarily involved the Line Director (i.e. head) of the Communicable Disease Control Program (which is within the Directorate General of Health Services, an agency of the Ministry of Health and Family Welfare), which is responsible for the national Antimicrobial Resistance Containment programme, and the District Livestock Officer of Cumilla district (who has overall responsibility for government-related activities in relation to livestock issues within the district, as part of the Ministry of Fisheries and Livestock). These meetings ultimately led to a memorandum of understanding being signed where it was agreed that the Ministry of Health and Family Welfare and the Ministry of Fisheries and Livestock would support the research as described below.

### 3.3.2. Training of master trainers

Next, the Bangladeshi research team led a three-day training programme in Dhaka for the master trainers, who were national-level government and academic specialists in AMR, One Health and/or community engagement, and who would subsequently train the trainers. All master trainers had to have 1) extensive contextualised knowledge and working expertise on AMR and related fields (e.g. human/animal health), 2) experience conducting training sessions/capacity development in general (ideally including experience of developing contextualised training modules) and in relation to AMR, 3) experience of community engagement. The goals of the training programme were as follows. 1) To provide the master trainers with all necessary information about AMR as related to the focus of the CDs. 2) To familiarise master trainers with the tools and procedures developed to support the CDs. 3) To co-develop, validate and finalise the CD toolkit (facilitator guide and flipchart plus supervision and monitoring tools – see below). 4) To familiarise master trainers with the monitoring and supervision process and tools that will be used to monitor the CDs and help supervise the facilitators. 5) To prepare the master trainers to train the trainers.

These master trainers are listed in table S2. The master trainers were selected based on discussions between the Bangladeshi research team and senior officials in both the Bangladeshi government’s Communicable Disease Control Program and the Ministry of Fisheries and Livestock. The training programme was supported by a manual developed by the research team, which described the content and format of the training programme. The training consisted of a mixture of lectures and role play activities.

| **Table S2. Master trainers.** | |
| --- | --- |
| **Professional title** | **Job role** |
| **Human health related** | |
| Medical Officer (Communicable Disease Control, Directorate General Health Services, Ministry of Health and Family Welfare) | Medical Officer working on the National Action Plan: Antimicrobial Resistance Containment in Bangladesh, and in the Viral Hepatitis & Diarrhoeal Disease unit of Communicable Disease Control Program, and on implementing other activities of the Communicable Disease Control Program. |
| Evaluator (Communicable Disease Control, Directorate General Health Services, Ministry of Health and Family Welfare) | Programme evaluator working on the National Action Plan: Antimicrobial Resistance Containment in Bangladesh, and in the Viral Hepatitis & Diarrhoeal Disease unit of Communicable Disease Control Program, and on other activities of the Communicable Disease Control Program. |
| **Animal health related** | |
| Animal Health Expert (Chattogram Veterinary and Animal Sciences University) | Involved in research and teaching on Animal Health and One Health. |
| District Livestock Officer (Cumilla) | Overall responsibility for government-related activities in relation to livestock issues at a district level. |
| Assistant Professor (Dhaka and Mugda Medical College) | Involved in teaching on, and subject expert on, microbiology and AMR. |

### 3.3.3. District-level sensitisation meeting

Next, the Bangladeshi research team held a sensitisation meeting with national-level, district-level and subdistrict-level governmental stakeholders at Cumilla district’s Civil Surgeon Office. The stakeholders who attended this meeting are listed in table S3. The two goals were 1) to explain the rationale and importance of the project and to seek these stakeholders’ specific support with conducting the intervention activities, and 2) to compile a list of potential trainers to support the intervention delivery (as explained more below). In relation to goal 2, the Bangladeshi research team and representatives from the Communicable Disease Control Program requested that in each subdistrict the subdistrict’s Upazila Health Family Planning Officer (the head of the subdistrict’s Upazila Health Complex, which are subdistrict-level primary care hospitals) along with the District Livestock Officer (the government official responsible for overseeing and managing livestock related activities and issues at the district level that are the government’s responsibility) suggested two trainers from the human health sector (either Medical Officers or Assistant Surgeons from the subdistrict’s Upazila Health Complex) and two trainers from the animal health sector (either Livestock Extension Officers or Upazila Livestock Officers from the Upazila Livestock Office [subdistrict-level government office responsible for overseeing and managing livestock related activities and issues at the district level that are the government’s responsibility]) for each study subdistrict.

| **Table S3. District-level sensitisation meeting stakeholders** | |
| --- | --- |
| **Stakeholders** | **Job role** |
| **Human health related** | |
| Deputy Program Manager (Communicable Disease Control, Directorate General Health Services, Ministry of Health and Family Welfare) | Deputy Program Manager for the National Action Plan: Antimicrobial Resistance Containment in Bangladesh, and the Viral Hepatitis & Diarrhoeal Disease unit of Communicable Disease Control Program. Also oversees and implements other activities of the Communicable Disease Control Program. |
| Civil Surgeon (Cumilla district) | Overall responsibility for the human health system and human health at a district level. |
| Upazila Health and Family Planning Officers (from Barura, Burichang, Brahmanpara, Homna, and Daudkandi subdistricts) | Head of their subdistrict’s Upazila Health Complex^a^ |
| Resident Medical Officers (from Barura, Burichang, Brahmanpara, Homna, and Daudkandi subdistricts) | Registered healthcare provider at the Upazila Health Complex^a^ |
| Assistant Surgeons (from Barura, Burichang, Brahmanpara, Homna, and Daudkandi subdistricts) | Registered healthcare provider at the Upazila Health Complex^a^ |
| Medical Officers (from Barura, Burichang, Brahmanpara, Homna, and Daudkandi subdistricts) | Registered healthcare provider at the Upazila Health Complex^a^ |
| **Animal health related** | |
| Veterinary Surgeons (from Barura, Burichang, Brahmanpara, Homna, and Daudkandi subdistricts) | Registered animal healthcare provider |
| Upazila Livestock Officers (from Barura, Burichang, Brahmanpara, Homna, and Daudkandi subdistricts) | Head of their subdistrict’s Upazila Livestock Office^b^ and Veterinary Hospital |
| Livestock Extension Officers (from Barura, Burichang, Brahmanpara, Homna, and Daudkandi subdistricts) | Registered animal healthcare provider |
| District Livestock Officer (Cumilla district) | Overall responsibility for government-related activities in relation to livestock issues at a district level. |
| **Political** | |
| Upazila Chairman | Subdistrict-level elected government representative |
| ^a^ Upazila Health Complexes are government-run rural hospitals offering in-patient and out-patient care.  ^b^ Subdistrict-level government office responsible for overseeing and managing livestock related activities and issues at the district level that are the government’s responsibility | |

### 3.3.4. Training of trainers

Next, the master trainers, supported by members of the Bangladeshi research team, conducted a three-day-long training programme at the Civil Surgeon Office in Cumilla to train the trainers nominated during the district-level sensitisation meeting. The main goal of this programme was to train the trainers so that they could ultimately train the facilitators and their supervisors, but a secondary goal was to build the capacity of the subdistrict-level healthcare providers from the human and animal sectors on AMR from a One Health perspective and on community engagement, to facilitate eventual scale-up and sustainability of the intervention. The training programme was supported by a manual developed by the research team, which described the content and format of the training programme. The training consisted of a mixture of lectures and role play activities.

### 3.3.5. Sensitisation of subdistrict-level and lower-level stakeholders

Next, following randomisation of clusters in each subdistrict (see *Trial schedule*) sensitisation meetings were held in each subdistrict with local (subdistrict-level/community-level) stakeholders from the human health, animal health, general civil service and political sectors. The two goals of these meetings were to 1) seek local stakeholders’ approval and support for the project (see table S4 and subsequent sub-sections for details of their involvement), and 2) to sensitise the supervisors about their role in selecting the facilitators. These sensitisation discussions were supported by information sheets describing the study.

| **Table S4. Subdistrict-level sensitisation meetings stakeholder list** | | | | |
| --- | --- | --- | --- | --- |
| **Stakeholders** | **Job role** | **Job location** | **Intervention role** |  |
| **Human health related** | | | |  |
| Upazila Health and Family Planning Officer | Head of Upazila Health Complex^a^ | Upazila Health Complex^a^ | Trainer and provided overall administrative support |  |
| Sanitary Inspector | Sanitary inspection for the subdistrict | Upazila Health Complex^a^ | Stakeholder providing a One Health perspective on AMR, particularly from an environment perspective |  |
| Assistant Surgeon | Registered healthcare provider | Upazila Health Complex^a^ | Trainer |  |
| Medical Officer | Registered healthcare provider | Upazila Health Complex^a^ | Trainer |  |
| Community Health Care Provider (CHCP) | Basic human healthcare provider | Community Clinic | Supervisor of CD facilitators |  |
| Assistant Health Inspector | Supervisor of CHCP and Health Assistant | Union | Supervisor of CD facilitators |  |
| Health Assistant | Basic healthcare provider and responsible for ward-level Expanded Immunization Program | Ward | Supervisor of CD facilitators |  |
| **Animal health related** | | | |  |
| Upazila Livestock Officer | Head of Upazila Livestock Office and Veterinary Hospital | Upazila Livestock Office | Trainer |  |
| Veterinary Surgeon | Registered animal healthcare provider | Upazila Livestock Office | Trainer |  |
| Livestock Extension Officer | Registered animal healthcare provider | Upazila Livestock Office | Trainer |  |
| **Civil service** |  |  |  |  |
| Upazila Nirbahi Officer | Chief government administrative officer for the subdistrict | Upazila Administration | Stakeholder and subdistrict-level gatekeeper: conducting any project activities in a subdistrict requires the permission of their office. |  |
| Upazila Education Officer | Education officer | Upazila Education Office | Stakeholder. As community dialogue is a iterative educative process, in some sub-districts, Upazila Education Officer were invited and sensitised about this approach. |  |
| **Political** | | | |  |
| Union Parishad Chairman | Elected chairman (local government representative) of each union (the administrative level below a subdistrict in Bangladeshi local government) | Union Parishad | Stakeholder and cluster-level gatekeeper: conducting any project activities in a village/villages requires the permission of their office. They also assisted the data collectors in accessing villages/households for the baseline and endline surveys, helped to mobilise community members when recruiting facilitators, and helped to mobilise community members to attend the CDs. |  |
| ^a^ Upazila Health Complexes are government-run rural hospitals offering in-patient and out-patient care. | | | | |

### 3.3.6. Selection of supervisors and facilitators

During the training of trainers programme it was decided between the Bangladeshi research team and the Upazila Health and Family Planning Officers that all facilitators would be supervised by one supervisor: either the health care provider for the cluster’s Community Clinic (the Community Health Care Practitioner) or the Health Assistant/Assistant Health Inspector associated with the Community Clinic (see table S4). It was also decided that two supervisors would be selected in each intervention cluster to share the supervision duties for all the facilitators in that cluster. These supervisors had therefore attended the previous subdistrict-level sensitisation meetings, where they were also provided with information sheets about the study and their proposed roles.

Based on formative research and local knowledge of the cultural context, it was expected that female community members would be reluctant to attend CDs facilitated by men but would be more inclined to attend CDs facilitated by women. Consequently, the Bangladeshi research team decided that in each intervention cluster in every village we would aim to have at one female and one male facilitator, so that a parallel set of female-facilitated and male-facilitated CD sessions could be run in each village.

Consequently, in each intervention cluster within each village the relevant Union Parishad Chairman (see table S4) helped mobilise community members to apply to become facilitators. To be eligible to apply for the facilitator role an individual had to be aged 18 or older, have at least secondary-level education, be of good standing in the community, and be comfortable communicating with community members. However, the supervisors who had been previously selected for each cluster then selected the facilitators for each village from among the eligible volunteers on a largely subjective basis. Those who were selected were then invited to a training programme along with their supervisors.

### 3.3.7. Training of facilitators and supervisors

Next, within each subdistrict the facilitators and supervisors from all the intervention clusters in that subdistrict then attended a joint training programme at the subdistrict’s Upazila Health Complex. For the facilitators this programme lasted for three days but the supervisors only attended the first and last days. The goals of this programme were as follows. 1) To introduce the facilitators and supervisors to key information about AMR from a One Health perspective related to the CDs. 2) To familiarise the facilitators with the supporting materials (facilitator guide and flipchart – see below). 3) To strengthen facilitators’ communication skills. 4) To familiarise the facilitators and supervisors with the CD monitoring and supervision tools. 5) To start the planning process for running regular CDs and establishing the monthly supervision meetings, as per the intended schedule.

During the training programme every facilitator was provided with a guide, developed by the research team, that summarised the intended goals of the CDA, the intended format of CD sessions and how to facilitate them, and the intended roles and responsibilities of a facilitator. They were also provided with a flipchart, again developed by the research team, and trained how to use it to support their facilitation of the CDs (see below). The programme was delivered by the trainers, with support from members of the Bangladeshi research team, and supported by a manual developed by the research team, which described the content and format of the training programme. The training consisted of a mixture of lectures and role play activities.

## 3.4. Implementation and delivery of Community Dialogues

### 3.4.1. Community Dialogue delivery structure

The Bangladeshi research team decided that within each intervention cluster in each village each female and male facilitator will be asked (via their training and supervision) to aim to deliver 11 different CDs. Each CD will cover different specific aspects of AMR from a One Health perspective, with a focus on community-level and individual-level issues in the rural Bangladeshi context. The information and key messages to be delivered in each CD were developed by the research team, and were specified via the training, facilitator guide and flipchart. The flipchart provided simple pictorial illustrations of the key messages to help facilitators communicate the ideas behind the key messages in a simple and effective way, even when attendees had low literacy levels or were illiterate. See table S5 for each session’s key messages. Facilitators will be trained to aim for each CD to take no longer than 90 minutes, and where possible to be delivered to 20-30 community members. The facilitators will be asked to aim to deliver each of the 11 CDs twice, approximately two-weeks apart, at separate areas in their village, to maximise coverage and participation. The aim is therefore to deliver each distinct CD twice per month in each village for one year (with no CDs during the month of Ramadan). It is planned that the facilitator will organise the location for the CD but supported by their local Union Parishad Chairman, and that this should be a public space like a courtyard or school building. Prior to facilitating their first CD, it has also been planned that all facilitators will meet with their supervisors to plan and prepare for this first session.

### 3.4.2. Facilitator supervision and Community Dialogue monitoring and evaluation

The Bangladeshi research team also developed a monitoring and evaluation process for the facilitators to report on their CDs. At the end of each CD the facilitators should seek feedback from the community members via a score book. Then, after each CD the facilitators should meet with their supervisor to review how the meeting went, discuss the feedback and any issues and how to overcome them. In this meeting they should complete a brief report about the CD and a decision log to record any decisions the community made in the CD. The supervisors will be provided with a supervision checklist that lists the issues they should discuss with the facilitator. Finally, the facilitators will be supported to plan for their next CD by their supervisor and by a monthly CD plan template. In addition, field officers will attend the supervisory meetings on an ad-hoc basis to ensure that the CDs are running effectively and to help solve any issues. They will also help to maintain communication between the supervisors and facilitators. Their role is to act as a bridge between the facilitators and supervisors and the Bangladeshi research team and will be recruited by the Bangladeshi research team.

The Bangladeshi research team has also planned that every quarter all facilitators will be asked to participate in a quarterly feedback meeting and refresher training process, where the supervisors will also be present. This will be held at each subdistrict’s Upazila Health Complex and is expected to take about three hours. The goal of the feedback portion of the meetings will be to gather feedback on the experiences of facilitators, review the monitoring process and explore whether any facilitators need any further support. The goal of the refresher training portion will be to refresh facilitators’ understanding about how the research team want the CDs to be managed and delivered and how the reporting processes should work. The meetings will be run by members of the Bangladeshi research team supported by the trainers where possible, and the supervisors will be present so they can then undertake any required actions during their subsequent meetings with facilitators.

# 4. Survey questionnaire

Note that the below questionnaire is the endline survey questionnaire, but this contains the same questions as the baseline survey questionnaire plus some additional questions that are self-evident.

|  | **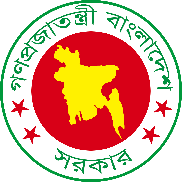** |  |
| --- | --- | --- |
| **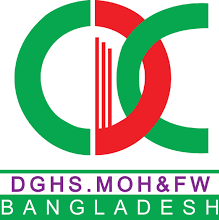** |  | **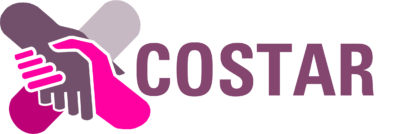** |
|  |  |  |

Engaging rural communities in Bangladesh to address antimicrobial resistance via the Community Dialogue Approach: a protocol for a cluster-randomised controlled trial

**Nature of the Survey:** Household Survey

**Respondent Nature:** Community People from Barura, Brahmanpara, Burichang, Daudkandi and Homan Upazilla of Cumilla District


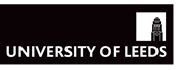


**
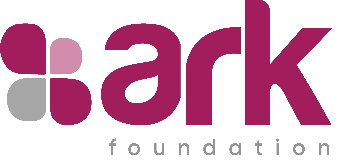
**

**Basic survey interview information**

| **No** | **Questions** | **Coding categories** | **Code** | **Skip** |
| --- | --- | --- | --- | --- |
| **I** | Date (dd/mm/yyyy) | \| **D** \| **D** \| **M** \| **M** \| **Y** \| **Y** \| **Y** \| **Y** \| \| --- \| --- \| --- \| --- \| --- \| --- \| --- \| --- \| |  |  |
| II | Interviewer name | Xxxx |  |  |
| III | Interviewer ID |  |  |  |
| IV | Survey district | Cumilla |  |  |
| V | Subdistrict | \|  \|  \| \| --- \| --- \| |  |  |
| VI | Union | \|  \|  \| \| --- \| --- \| |  |  |
| VII | Village | *Select the Village name from the drop-down list*  *______________* |  |  |
| VIII | Community clinic | CC_name  *(Select the CC name from the drop-down list)* |  |  |
| IX | Survey round | Endline | 1 |  |
| ***ELIGIBILITY Criteria***  ***Ask the respondent from the Interviewed Household if they took part in the COSTAR Baseline Survey.*** | | | | |
| XI | Did you take part in the COSTAR baseline survey? | YES |  | End the survey. |
|  |  | NO |  | Proceed with the interview |
|  |  | YES but proceed with the interview due to lack of respondents in that village. |  | Proceed with the interview |
|  |  |  |  |  |
| XII | Interview result | Completed | 1 |  |
|  |  | No person or no eligible person at household for interview | 2 | **END** |
|  |  | Postponed  (Record date of planned return/ Reschedule)   \| **D** \| **D** \| **M** \| **M** \| **Y** \| **Y** \| **Y** \| **Y** \| \| --- \| --- \| --- \| --- \| --- \| --- \| --- \| --- \|   (Record time of planned return/ Reschedule)   \|  \|  \| \| --- \| --- \|   (am/pm) | 3 | **END** |
|  |  | Refused | 4 | **END** |
|  |  | Other (specify)_______________ | 555 |  |
| XIII | Latitude | \|  \| \| --- \| |  |  |
| XIV | Longitude | \|  \| \| --- \| |  |  |
| XV | Interview start time | \|  \|  \| \| --- \| --- \|   (am/pm) |  |  |
| XVI | Interview end time | \|  \|  \| \| --- \| --- \|   (am/pm) |  |  |
| XVII | Do you agree to take part in the study? | Yes | 1 | **→ 1** |
|  |  | No | 2 | **End** |
| XVIII | Household holding number | \|  \|  \|  \|  \| \| --- \| --- \| --- \| --- \| |  |  |

| ***Household and respondent characteristics variables*** | | | | | | |
| --- | --- | --- | --- | --- | --- | --- |
| **SL** | **Questions** | | **Coding categories** | | **Code** | **Skip** |
| 1 | Gender | | Male | | 1 |  |
|  |  |  | Female | | 2 |  |
|  |  |  | Other *(please specify_____)* | | 555 |  |
| 2 | How old are you? | | \|  \|  \| \| --- \| --- \|   *(in completed years)* | |  |  |
| 3 | How many family members currently live in your household? | | \|  \|  \| \| --- \| --- \|   *(in numbers)* | |  |  |
| 4 | What is your religion? | | Muslim | | 1 |  |
|  |  |  | Hindu | | 2 |  |
|  |  |  | Christian | | 3 |  |
|  |  |  | Buddhist | | 4 |  |
|  |  |  | Others  (Please specify) | | 555 |  |
| 5 | What is your last level of education you have had at an educational institution? | | No formal education | | 1 |  |
|  |  |  | Less than primary/equivalent | | 2 |  |
|  |  |  | Primary completed/equivalent | | 3 |  |
|  |  |  | Less than secondary/equivalent | | 4 |  |
|  |  |  | Secondary completed/equivalent | | 5 |  |
|  |  |  | Less than higher secondary/equivalent | | 6 |  |
|  |  |  | Higher secondary/equivalent | |  |  |
|  |  |  | Graduation/equivalent completed | | 7 |  |
|  |  |  | Post-graduation and above | | 8 |  |
| 6 | Have you done any work for cash or in-kind payment in the last 30-days? | | Yes | | 1 |  |
|  |  |  | No | | 2 | **→ 8** |
| 7 | What type of work was this, and if you have done more than one type of work, please just tell me the type you did most often? | | Work as a share cropper | |  |  |
|  |  |  | Day/unskilled laborer (domestic, agricultural) | |  |  |
|  |  |  | Skilled worker (long term contracted laborer) | |  |  |
|  |  |  | Own business | |  |  |
|  |  |  | Rickshaw/van puller | |  |  |
|  |  |  | Government Service holder/professionals | |  |  |
|  |  |  | Private Service holder/professional | |  |  |
|  |  |  | Farm owner_agro, fisheries, chicken, duck, cow | |  |  |
|  |  |  | Tailor | |  |  |
|  |  |  | Teacher | |  |  |
|  |  |  | Tuition | |  |  |
|  |  |  | Garment worker | |  |  |
|  |  |  | Restaurant worker | |  |  |
|  |  |  | Electrician | |  |  |
|  |  |  | Mechanic | |  |  |
|  |  |  | Travelling salesperson | |  |  |
|  |  |  | Barber | |  |  |
|  |  |  | Weaver/ Carpenter/ Painter | |  |  |
|  |  |  | Immigrant | |  |  |
|  |  |  | Retired Person | |  |  |
|  |  |  | Shopkeeper | |  |  |
|  |  |  | Handcraft-Seller | |  |  |
|  |  |  | Driver (Auto/Pickup/Leguna/CNG/Microbus/Bus) | |  |  |
|  |  |  | Others  *(Please specify_________)* | | 555 |  |
|  |  | |  | |  |  |
| ***Animal ownership*** | | | | | | |
|  |  | |  | |  |  |
| 8 | Do you or your family currently own any poultry(chicken, duck, pigeon, quail), cattle, goats, sheep or fish for producing food, or to eat them, or to sell or trade? | | Yes | | 1 |  |
|  |  |  | No | | 2 | **→ 12__n_a** |
| 9 | Do you or your family currently own any poultry (chicken, duck, pigeon, quail), for producing food, or to eat them, or to sell or trade? | | Yes | | 1 |  |
|  |  |  | No | | 2 |  |
| 10 | Do you or your family currently own any cattle for producing food, or to eat them, or to sell or trade? | | Yes | | 1 |  |
|  |  |  | No | | 2 |  |
| 11 | Do you or your family currently own any goats/sheep for producing food, or to eat them, or to sell or trade? | | Yes | | 1 |  |
|  |  |  | No | | 2 |  |
| 12 | Do you or your family currently own any fish to eat them, or to sell or trade? | | Yes | | 1 |  |
|  |  |  | No | | 2 |  |
|  |  | |  | |  |  |
| 12__n_a | Have you heard of any gatherings in your village or other villages that have been organised for people to discuss medicines and health? | | Yes | | 1 |  |
|  |  |  | No | | 2 | **→ 13** |
|  |  |  | Don’t Know | | -99 | **→ 13** |
| 12__n_b | From where did you hear about CDs?  Or  Please tell all the different places that you have heard about CDs. | | Family | | 1 |  |
|  |  |  | Friends | | 2 |  |
|  |  |  | CD Facilitators | | 3 |  |
|  |  |  | CHCPs/HAs/FWA/ health service providers | | 4 |  |
|  |  |  | Other community members | | 5 |  |
|  |  |  | Others (Specify) | | 555 |  |
| 12_n_c | Have you attended any of these discussions yourself? | | Yes | | 1 |  |
|  |  |  | No | |  | **→12_n_e** |
|  |  |  | Unsure | |  | **→12_n_e** |
| 12_n_d | How many of these discussions have you attended?  *(Mention the number of CDs attended as best as the respondent recall)* | | 0-22 | |  |  |
| 12_n_e | Have any of your family members attended any of these discussions? | | Yes | | 1 |  |
|  |  |  | No | | 2 |  |
|  |  |  | Don’t Know | | -99 |  |
| 12_n_f | Have any of your friends attended any of these discussions? | | Yes | | 1 |  |
|  |  |  | No | | 2 |  |
|  |  |  | Don’t Know | | -99 |  |
|  |  |  |  | |  |  |
| ***Outcomes*** | | | | | | |
|  |  | |  | |  |  |
| ***Primary outcome 1. Reported awareness of antibiotics as a type of medicine*** | | | | | | |
|  |  | |  | |  |  |
| 13 | Have you ever heard of a type of medicine known as an antibiotic or antibiotics? | | Yes | | 1 |  |
|  |  |  | No | | 2 | **→ 60** |
|  |  |  | Don’t know | | -99 | **→ 60** |
|  |  | |  | |  |  |
| ***Primary outcome 2. Correct knowledge about antibiotics and their appropriate use in relation to human health*** | | | | | | |
|  |  | |  | |  |  |
| ***General knowledge about antibiotics*** | | | | | | |
|  |  | |  | |  |  |
| 14 | Can antibiotics treat any illness? | | Yes | | 1 |  |
|  |  |  | No | | 2 |  |
|  |  |  | Don’t know | | -99 |  |
| 15 | Can antibiotics treat illnesses caused by viruses? | | Yes | | 1 |  |
|  |  |  | No | | 2 |  |
|  |  |  | Don’t know | | -99 |  |
| 16 | Can antibiotics treat illnesses caused by bacteria? | | Yes | | 1 |  |
|  |  |  | No | | 2 |  |
|  |  |  | Don’t know | | -99 |  |
| 17 | Can antibiotics treat illnesses not caused by viruses or bacteria or other microbes, like diabetes or high blood pressure (also called hypertension)? | | Yes | | 1 |  |
|  |  |  | No | | 2 |  |
|  |  |  | Don’t know | | -99 |  |
| 18 | Is there just one type of antibiotic? | | Yes | | 1 |  |
|  |  |  | No | | 2 |  |
|  |  |  | Don’t know | | -99 |  |
| 19 | Can antibiotics be given as a tablet? | | Yes | | 1 |  |
|  |  |  | No | | 2 |  |
|  |  |  | Don’t know | | -99 |  |
| 20 | Can antibiotics be given as an injection? | | Yes | | 1 |  |
|  |  |  | No | | 2 |  |
|  |  |  | Don’t know | | -99 |  |
| 21 | Can antibiotics be given as a cream/ointment? | | Yes | | 1 |  |
|  |  |  | No | | 2 |  |
|  |  |  | Don’t know | | -99 |  |
| 22 | Are more expensive antibiotics always better at treating illness than less expensive antibiotics? | | Yes | | 1 |  |
|  |  |  | No | | 2 |  |
|  |  |  | Don’t know | | -99 |  |
|  |  | |  | |  |  |
| ***Knowledge and attitudes related to antibiotic use*** | | | | | | |
|  |  | |  | |  |  |
| 23 | Assume that you became ill and when you went to get treatment from a drug seller, they recommended that you buy some antibiotics from them. Would you trust their advice and buy the antibiotics recommended or not trust their advice and not buy the antibiotics from them? | | Trust their advice | | 1 |  |
|  |  |  | Not trust their advice | | 2 |  |
|  |  |  | Don’t know | | -99 |  |
| 24 | Assume that you became ill and thought you needed antibiotics to get better and so you went to a qualified healthcare provider for treatment, such as a CHCP at your local community clinic, or a doctor at Upazila Health Complex or a qualified doctor at a private clinic. If they told you that you did not need antibiotics, would you trust their advice or ignore their advice and seek antibiotics elsewhere? | | Trust their advice | | 1 |  |
|  |  |  | Not trust their advice | | 2 |  |
|  |  |  | Don’t know | | -99 |  |
| 25 | Is it appropriate or inappropriate to buy antibiotics to keep at home in case you or someone else in the household gets ill in the future? | | Appropriate | | 1 |  |
|  |  |  | Inappropriate | | 2 |  |
|  |  |  | Don’t know | | -99 |  |
| 26 | If you or someone in your household gets ill and is treated with antibiotics and some are left over, is it appropriate or inappropriate to keep those antibiotics at home in case you or someone else in the household gets ill in the future? | | Appropriate | | 1 |  |
|  |  |  | Inappropriate | | 2 |  |
|  |  |  | Don’t know | | -99 |  |
| 27 | Assume that you are taking antibiotics for an illness and you start to feel better after taking just half the course you were advised to take. Is it appropriate or inappropriate to not take any of the rest of the course? | | Appropriate | | 1 |  |
|  |  |  | Inappropriate | | 2 |  |
|  |  |  | Don’t know | | -99 |  |
| 28 | Assume that you are ill and have been prescribed a course of antibiotics by a qualified healthcare provider, such as a community health care provider (CHCP) or a doctor at the Upazilla Health Complex or a qualified doctor at a private clinic, which you have been taking. If the antibiotics don’t seem to be helping do you think it is appropriate or inappropriate to take more antibiotics each day than advised without speaking to the provider first? | | Appropriate | | 1 |  |
|  |  |  | Inappropriate | | 2 |  |
|  |  |  | Don’t know | | -99 |  |
| 29 | Assume that you are ill and have been prescribed a course of antibiotics by a qualified healthcare provider, such as a community health care provider (CHCP) or doctor at the Upazilla Health Complex or a qualified doctor at a private clinic. Is it appropriate or inappropriate to keep the prescription for use again in the future if you had the same symptoms? | | Appropriate | | 1 |  |
|  |  |  | Inappropriate | | 2 |  |
|  |  |  | Don’t know | | -99 |  |
|  |  | |  | |  |  |
| ***Antibiotic resistance*** | | | | | | |
|  |  | |  | |  |  |
| 30 | Have you heard of any of the terms *“antibiotic resistance”*, *“antimicrobial resistance”* or *“drug resistance”*? | | Yes | | 1 |  |
|  |  |  | No | | 2 | **→38** |
|  |  |  | Don’t know | | -99 | **→38** |
| 30_n_a | From where did you first learn about the terms *“antibiotic resistance”*, *“antimicrobial resistance”* or *“drug resistance”*? | | From CDs | | 1 |  |
|  |  |  | From Family members | | 2 |  |
|  |  |  | From Friends | | 3 |  |
|  |  |  | From local community members | | 4 |  |
|  |  |  | From CHCP/HA/AHI/Other Local Healthcare service provider | | 5 |  |
|  |  |  | From Registered MBBS Doctor | | 6 |  |
|  |  |  | From Pharmacey/ Drug Store | | 7 |  |
|  |  |  | From posters in CC | | 8 |  |
|  |  |  | From Media (Electronic/Print/Social) | | 9 |  |
|  |  |  | Cannot Remember | | 10 |  |
|  |  |  | Don’t Know | | -99 |  |
|  |  |  | Others (Please Specify) | | 555 |  |
| 31 | Does antibiotic resistance happen when your body becomes resistant to an antibiotic? | | Yes | | 1 |  |
|  |  |  | No | | 2 |  |
|  |  |  | Don’t know | | -99 |  |
| 32 | Does antibiotic resistance happen when the microbes causing an infection become resistant to the antibiotic which can no longer kill them? | | Yes | | 1 |  |
|  |  |  | No | | 2 |  |
|  |  |  | Don’t know | | -99 |  |
| 33 | Can people who are not taking antibiotics get antibiotic resistant diseases, such as drug-resistant TB? | | Yes | | 1 |  |
|  |  |  | No | | 2 |  |
|  |  |  | Don’t know | | -99 |  |
| 34 | In Bangladesh, are antibiotic resistant diseases about as common now as in the past, or are they becoming less common or are they becoming more common? | | Same | | 1 |  |
|  |  |  | Less common | | 2 |  |
|  |  |  | More common | | 3 |  |
|  |  |  | Don’t know | | -99 |  |
| 35 | Which one do you think is easier to treat with antibiotics? An antibiotic resistant infection. An infection that is treatable with an antibiotic. Neither one is more treatable than the other. | | An antibiotic resistant infection | | 1 |  |
|  |  |  | An infection that is treatable with an antibiotic | | 2 |  |
|  |  |  | Neither one is more treatable than the other. | | 3 |  |
|  |  |  | Don’t know | | -99 |  |
| 36 | Can antibiotic resistant diseases spread from person to person? | | Yes | | 1 |  |
|  |  |  | No | | 2 |  |
|  |  |  | Don’t know | | -99 |  |
| 37 | Can vaccinations help prevent the diseases from occurring? | | Yes | | 1 |  |
|  |  |  | No | | 2 |  |
|  |  |  | Don’t know | | -99 |  |
|  |  | |  | |  |  |
| ***The importance of antibiotics*** | | | | | | |
|  |  | |  | |  |  |
| 38 | Can antibiotics save lives when used properly? | | Yes | | 1 |  |
|  |  |  | No | | 2 |  |
|  |  |  | Don’t know | | -99 |  |
|  |  | |  | |  |  |
| ***Primary outcome 3: Correct knowledge about antibiotics and their appropriate use in relation to animal health*** | | | | | | |
|  |  | |  | |  |  |
| ***Basic knowledge*** | | | | | | |
|  |  | |  | |  |  |
| 39 | Can antibiotics treat all illnesses of animals? | | Yes | | 1 |  |
|  |  |  | No | | 2 |  |
|  |  |  | Don’t know | | -99 |  |
| 40 | Can some diseases be spread between people and animals and between animals and people? | | Yes | | 1 |  |
|  |  |  | No | | 2 |  |
|  |  |  | Don’t know | | -99 |  |
| 41 | Can people get diseases that have become resistant to treatment by antibiotics from animals? | | Yes | | 1 |  |
|  |  |  | No | | 2 |  |
|  |  |  | Don’t know | | -99 |  |
| 42 | Assume you owned a cow that became ill and when you asked the local village doctor for help, they told you to give the cow some antibiotics. Would it be appropriate or inappropriate to follow their advice? | | Appropriate | | 1 |  |
|  |  |  | Inappropriate | | 2 |  |
|  |  |  | Don’t know | | -99 |  |
| 43 | Assume you owned a cow that became ill and you thought it needed antibiotics to get better, but when you went to the nearest upazila veterinary hospital you were told that antibiotics would not help. Would you trust their advice or seek antibiotics elsewhere? | | Trust their advice | | 1 |  |
|  |  |  | Not trust their advice | | 2 |  |
|  |  |  | Don’t know | | -99 |  |
| 44 | Assume you owned a cow. Is it appropriate or inappropriate to buy antibiotics to keep inside the home in case the cow gets ill in the future? | | Appropriate | | 1 |  |
|  |  |  | Inappropriate | | 2 |  |
|  |  |  | Don’t know | | -99 |  |
| 45 | Assume you owned a cow. If the cow became ill and needed to be treated with antibiotics and some are left over, is it appropriate or inappropriate to keep those antibiotics inside home in case the cow gets ill in the future? | | Appropriate | | 1 |  |
|  |  |  | Inappropriate | | 2 |  |
|  |  |  | Don’t know | | -99 |  |
| 46 | Assume you were taking antibiotics for an illness but you also owned a cow that became ill with similar symptoms. Would it be appropriate or inappropriate to give some of your antibiotics to your animal? | | Appropriate | | 1 |  |
|  |  |  | Inappropriate | | 2 |  |
|  |  |  | Don’t know | | -99 |  |
| 47 | Assume you owned a cow that became ill and you were treating it with antibiotics, and you also became ill with similar symptoms. Would it be appropriate or inappropriate to take some of your cow’s antibiotics for your own illness? | | Appropriate | | 1 |  |
|  |  |  | Inappropriate | | 2 |  |
|  |  |  | Don’t know | | -99 |  |
| 48 | Assume you owned a cow that became ill and you were advised to treat it with antibiotics by a qualified vet, such as a Upazilla livestock officer or a livestock extension officer from a Upazila veterinary hospital, and the animal seemed to recover after you gave it just half the course you were advised to give it. Would it be appropriate or inappropriate to stop treating it and not give it the rest of the course? | | Appropriate | | 1 |  |
|  |  |  | Inappropriate | | 2 |  |
|  |  |  | Don’t know | | -99 |  |
| 49 | Assume you owned a cow that became ill and you were advised to treat it with antibiotics by a qualified vet, such as a Upazilla livestock officer or a livestock extension officer from a Upazila veterinary hospital. If the antibiotics didn’t seem to be helping, would it be appropriate or inappropriate to give the cow more of the antibiotics each day than you were advised to without speaking to the vet again first? | | Appropriate | | 1 |  |
|  |  |  | Inappropriate | | 2 |  |
|  |  |  | Don’t know | | -99 |  |
|  |  | |  | |  |  |
| ***Non-disease uses of antibiotics in animals*** | | | | | | |
|  |  | |  | |  |  |
| 50 | Can giving healthy animals antibiotics, for example in their feed, to make them grow better or produce more milk or eggs, cause antibiotic resistant infections to happen in the animals? | | Yes | | 1 |  |
|  |  |  | No | | 2 |  |
|  |  |  | Don’t know | | -99 |  |
| 51 | Can giving healthy animals antibiotics, for example in their feed or water, to try and stop them from getting any diseases they don’t currently have in the future, cause antibiotic resistant infections to happen in the animals, which can be hard to treat with antibiotics? | | Yes | | 1 |  |
|  |  |  | No | | 2 |  |
|  |  |  | Don’t know | | -99 |  |
|  |  | |  | |  |  |
| ***Antibiotic contamination in relation to animals*** | | | | | | |
|  |  | |  | |  |  |
| 52 | If an animal is given an antibiotic and someone eats the animals’ meat, eggs or milk within a few days of the animal being given the antibiotic can the antibiotic be passed into their body by eating the animal’s meat, eggs or milk? | | Yes | | 1 |  |
|  |  |  | No | | 2 |  |
|  |  |  | Don’t know | | 3 |  |
| 53 | If an animal is given an antibiotic does the antibiotic also get spread into the environment (for example into ponds or the soil) in the animal’s faeces and urine? | | Yes | | 1 |  |
|  |  |  | No | | 2 |  |
|  |  |  | Don’t know | | -99 |  |
| 54 | Can you help to stop the spread of antibiotic resistant infections by keeping animals away from open water sources, like ponds? | | Yes | | 1 |  |
|  |  |  | No | | 2 |  |
|  |  |  | Don’t know | | -99 |  |
| 55 | Can you help to stop the spread of antibiotic resistant infections by not giving healthy animals feed containing antibiotics? | | Yes | | 1 |  |
|  |  |  | No | | 2 |  |
|  |  |  | Don’t know | | -99 |  |
|  |  | |  | |  |  |
| ***Vaccination*** | | | | | | |
|  |  | |  | |  |  |
| 56 | Can vaccinating animals stop them from getting certain infectious diseases? | | Yes | | 1 |  |
|  |  |  | No | | 2 |  |
|  |  |  | Don’t know | | -99 |  |
| 57 | Can vaccinating animals stop them from spreading diseases, including antibiotic resistant diseases, to humans? | | Yes | | 1 |  |
|  |  |  | No | | 2 |  |
|  |  |  | Don’t know | | -99 |  |
|  |  | |  | |  |  |
| ***Antibiotic importance*** | | | | | | |
|  |  | |  | |  |  |
| 58 | Can antibiotics ever save animals’ lives if used properly (according to the vet doctors advise)? | | Yes | | 1 |  |
|  |  |  | No | | 2 |  |
|  |  |  | Don’t know | | -99 |  |
|  |  | |  | |  |  |
| **Questions related to secondary outcomes on reported-practices** | | | | | | |
|  |  | |  | |  |  |
| ***Household member recent illness with treatment seeking*** | | | | | | |
|  |  | |  | |  |  |
| 59 | Within the last three months have you shared any antibiotics that you already had, for example leftover from a previous illness, with any family, friends or neighbours? | | Yes | | 1 |  |
|  |  |  | No | | 2 |  |
|  |  |  | Don’t know | | -99 |  |
| 60 | Within the last three months have you been ill and sought treatment for any illnesses? | | Yes | | 1 |  |
|  |  |  | No | | 2 | **→ 72** |
|  |  |  | Don’t know | | -99 | **→ 72** |
| 61 | Within the last three months how many times have you been ill and sought treatment for those illnesses? | | *Write down the number of ill episodes in number*  *____________________* | |  |  |
|  |  | |  | |  |  |
| ***I will now ask you about the most recent illness episode within the last three months where you have sought treatment for that illness.*** | | | | | | |
|  |  | |  | |  |  |
| ***For the most recent illness episode (most recent or only illness)*** | | | | | | |
|  |  | |  | |  |  |
| 62 | What was the type of illness? | | Chest cold | |  |  |
|  |  |  | Common cold | |  |  |
|  |  |  | Ear infection | |  |  |
|  |  |  | Flu (influenza) | |  |  |
|  |  |  | Sinus infection (sinusitis) | |  |  |
|  |  |  | Skin infections | |  |  |
|  |  |  | Sore throat | |  |  |
|  |  |  | Urinary tract infections (bladder infection, kidney problem) | |  |  |
|  |  |  | TB | |  |  |
|  |  |  | Pneumonia | |  |  |
|  |  |  | Diarrhea | |  |  |
|  |  |  | Malaria | |  |  |
|  |  |  | Dengue | |  |  |
|  |  |  | HIV | |  |  |
|  |  |  | COVID-19 | |  |  |
|  |  |  | Cholera | |  |  |
|  |  |  | Diabetes | |  |  |
|  |  |  | Heart problem | |  |  |
|  |  |  | Gastric problem | |  |  |
|  |  |  | Hypertension | |  |  |
|  |  |  | Brain stroke related disease | |  |  |
|  |  |  | Renal disease | |  |  |
|  |  |  | Nervous and mental disease | |  |  |
|  |  |  | Arthritis and allied disease | |  |  |
|  |  |  | Bronchitis | |  |  |
|  |  |  | Asthma | |  |  |
|  |  |  | Cancer | |  |  |
|  |  |  | Chronic result of accidents | |  |  |
|  |  |  | Nasal polypus | |  |  |
|  |  |  | Surgery | |  |  |
|  |  |  | Thyroid disease | |  |  |
|  |  |  | Tumor | |  |  |
|  |  |  | Liver cirrhosis | |  |  |
|  |  |  | Lung problem | |  |  |
|  |  |  | Eye problem | |  |  |
|  |  |  | Fever | |  |  |
|  |  |  | Chikungunya | |  |  |
|  |  |  | Birth complications (pre/post/pregnancy) | |  |  |
|  |  |  | Stomach pain | |  |  |
|  |  |  | Weakness | |  |  |
|  |  |  | Arsenic | |  |  |
|  |  |  | Allergy | |  |  |
|  |  |  | Dysentery | |  |  |
|  |  |  | Chickenpox | |  |  |
|  |  |  | Measles | |  |  |
|  |  |  | Others  (Please specify_________) | | 555 |  |
| 63 | Where did you seek treatment for this illness? (If you went to more than one place to seek treatment for this illness, where did you go first?) | | CHCP in Community Clinic | |  |  |
|  |  |  | Doctor in Upazilla Health Complex | |  |  |
|  |  |  | Doctor in district level health complex | |  |  |
|  |  |  | Doctor in union health and family welfare Centre | |  |  |
|  |  |  | SACMO in UHC | |  |  |
|  |  |  | Dental Surgeon | |  |  |
|  |  |  | Doctor in specialized health complex | |  |  |
|  |  |  | Private medical practitioner | |  |  |
|  |  |  | Paramedic | |  |  |
|  |  |  | Village Doctor | |  |  |
|  |  |  | Health assistant | |  |  |
|  |  |  | Pharmacist | |  |  |
|  |  |  | Drug seller | |  |  |
|  |  |  | NGO medical facility | |  |  |
|  |  |  | Homeopathy doctor | |  |  |
|  |  |  | Others  (Please specify_________) | | 555 |  |
| 63_n_a | How much did you spend as the consultation fee? | | _______________ (BDT) | |  |  |
| 63_n_b | How much did you spend as ‘travel’ cost to reach the health facility from your house and vice versa? | | _______________ (BDT) | |  |  |
| 63_n_c | Did the doctor give you any diagnostic tests? | | Yes | | 1 | → 63_n_c |
|  |  |  | No | | 2 |  |
|  |  |  | Don’t know | | -99 |  |
| 63_n_d | If yes, how much did you spend for the diagnostic tests? | | _______________ (BDT) | |  |  |
|  |  | |  | |  |  |
|  |  | |  | |  |  |
| 64 | Did you take any antibiotics for this illness? | | Yes | | 1 |  |
|  |  |  | No | | 2 | **→ 72** |
|  |  |  | Don’t know | | -99 | **→ 72** |
| 64_n_a | What was the cost for the “antibiotics” medicine? | | _______________ (BDT) | |  |  |
| 64_n_b | Was the doctor/healthcare provider give you the antibiotics before or after the diagnostic tests? | | Before the diagnostic tests | | 1 |  |
|  |  |  | After the diagnostic tests | | 2 |  |
|  |  |  | Cannot remember | | 3 |  |
|  |  |  | Don’t Know | | -99 |  |
| 65 | Did you share any of these antibiotics with any other family members/friends who may have also been ill? | | Yes | | 1 |  |
|  |  |  | No | | 2 |  |
|  |  |  | Don’t know | | -99 |  |
| 66 | Where did you get the antibiotics from: 1) given them by a family member or a friend, 2) bought them (from a pharmacy for example), or 3) given them for free by a healthcare provider?  (Multiple answers are possible) | | Given by family/friend | | 1 | **→ 72** |
|  |  |  | Bought | | 2 |  |
|  |  |  | Free from provider | | 3 | **→ 69** |
|  |  |  | Don’t know | | -99 | **→ 72** |
| 67 | Where did you buy the antibiotics from? | | Pharmacy | |  |  |
|  |  |  | Local grocery shop | |  |  |
|  |  |  | Private medical practitioner/ Hospital | |  |  |
|  |  |  | Paramedic clinic | |  |  |
|  |  |  | Village Doctor Clinic | |  |  |
|  |  |  | NGO medical facility | |  |  |
|  |  |  | Other (please specify) | | 555 |  |
|  |  |  | Don’t know | | -99 |  |
| 67_a | Usually community clinic, upazilla health complex, District health complex, SACMO in UHC provide free medicine to the patients.  As you have taken treatment from Community Clinic/UHC/District Hospital/SACMO in UHC and Purchase antibiotic medicine from somewhere else  What are the reasons to buy antibiotic medicines from somewhere else? | | Doctor from the health facility advised to purchase medicine from outside | |  | If the answer to question **Q63** is any of the options among 1, 2, 3, 4, 5 and if the answer to question **Q66** is 2 or multiple options, where option 2 is included then the questioner will ask **Q67** and then **Q67_a** will apply. |
|  |  |  | Advised medicine from the doctor is not available at the health facility | |  |  |
|  |  |  | The medicine was not stocked in the health center | |  |  |
|  |  |  | The full course of medicine was not given by the health facility | |  |  |
|  |  |  | Others  (Please specify_________) | | 555 |  |
| 68 | When you bought the antibiotics did you use a prescription? | | Yes | | 1 |  |
|  |  |  | No | | 2 |  |
|  |  |  | Don’t know | | -99 |  |
| 69 | Which health care provider gave you the antibiotics | | CHCP in Community Clinic | |  |  |
|  |  |  | Doctor in Upazilla Health Complex | |  |  |
|  |  |  | Doctor in district level health complex | |  |  |
|  |  |  | Doctor in union health and family welfare Centre | |  |  |
|  |  |  | SACMO in UHC | |  |  |
|  |  |  | Dental Surgeon | |  |  |
|  |  |  | Doctor in specialized health complex | |  |  |
|  |  |  | Private medical practitioner | |  |  |
|  |  |  | Paramedic | |  |  |
|  |  |  | Village Doctor | |  |  |
|  |  |  | Health assistant | |  |  |
|  |  |  | Pharmacist | |  |  |
|  |  |  | Drug seller | |  |  |
|  |  |  | NGO medical facility | |  |  |
|  |  |  | Homeopathy doctor | |  |  |
|  |  |  | Others  (Please specify_________) | | 555 |  |
|  |  |  | Don’t know | | -99 |  |
|  |  |  |  | |  |  |
|  |  |  |  | |  |  |
| 70 | Did the provider give a prescription for the antibiotics? | | Yes | | 1 |  |
|  |  |  | No | | 2 |  |
|  |  |  | Don’t know | | -99 |  |
| 71 | Did you complete all the antibiotics that you got? | | Yes | | 1 |  |
|  |  |  | No | | 2 |  |
|  |  |  | Don’t know | | -99 |  |
|  |  | |  | |  |  |
|  |  | |  | |  |  |
| ***Family member most recent illness with treatment seeking*** | | | | | | |
|  |  | |  | |  |  |
| 71_n_a | How many children do you have? | | Open ended | | Numeric Field |  |
|  |  | |  | |  |  |
|  |  | |  | |  |  |
| 71_n_b | How many of your children are under 15 years at the time of interview | | Open ended | |  |  |
|  |  | | *The enumerator will put the number of the children in the open field.* | | Based on the number of responses in the open ended field, a separate number of profile will be create for each children. |  |
| 72 | Within the last three months have any of your family members aged under 15 been ill and sought treatment for the illness? | | Yes | | 1 |  |
|  |  |  | No | | 2 | **→ 85** |
|  |  |  | Don’t know | | -99 | **→ 85** |
|  |  |  |  | |  |  |
| 72_a | Within the last three months how many of your family members aged under 15 been ill and sought treatment for those illnesses? | | *Write down the number of ill Persons (in number)*  *____________________* | |  |  |
| ***For each family member under the age of 15 who has been ill within the last three months and sought treatment for the illness: I will now ask you about each of these family members and their most recent illnesses where they sought treatment in turn.*** | | | | | | |
|  |  | |  | |  |  |
| ***Family member ‘x’*** | | | | | | |
|  |  | |  | |  |  |
| 72_n_a | Name of the Children | | ____________ | | Text Field |  |
| 73 | Gender | | Male | | 1 |  |
|  |  |  | Female | | 2 |  |
|  |  | | Others  (please specify_____) | | 555 |  |
| 74 | How old is the family member ‘x’? | | \|  \|  \| \| --- \| --- \|   *(in completed years)* | |  |  |
| 75 | What is the last level of education you have had at an educational institution? | | No formal education | | 1 |  |
|  |  |  | Less than primary/equivalent | | 2 |  |
|  |  |  | Primary completed/equivalent | | 3 |  |
|  |  |  | Less than secondary/equivalent | | 4 |  |
|  |  |  | Secondary completed/equivalent | | 5 |  |
|  |  |  | Don’t know | | -99 |  |
|  |  | |  | |  |  |
| ***For their most recent illness*** | | | | | | |
|  |  | |  | |  |  |
| 76 | What was the type of illness? | | Chest cold | |  |  |
|  |  |  | Common cold | |  |  |
|  |  |  | Ear infection | |  |  |
|  |  |  | Flu (influenza) | |  |  |
|  |  |  | Sinus infection (sinusitis) | |  |  |
|  |  |  | Skin infections | |  |  |
|  |  |  | Sore throat | |  |  |
|  |  |  | Urinary tract infections (bladder infection, kidney problem) | |  |  |
|  |  |  | TB | |  |  |
|  |  |  | Pneumonia | |  |  |
|  |  |  | Diarrhea | |  |  |
|  |  |  | Malaria | |  |  |
|  |  |  | Dengue | |  |  |
|  |  |  | HIV | |  |  |
|  |  |  | COVID-19 | |  |  |
|  |  |  | Cholera | |  |  |
|  |  |  | Diabetes | |  |  |
|  |  |  | Heart problem | |  |  |
|  |  |  | Gastric problem | |  |  |
|  |  |  | Hypertension | |  |  |
|  |  |  | Brain stroke related disease | |  |  |
|  |  |  | Renal disease | |  |  |
|  |  |  | Nervous and mental disease | |  |  |
|  |  |  | Arthritis and allied disease | |  |  |
|  |  |  | Bronchitis | |  |  |
|  |  |  | Asthma | |  |  |
|  |  |  | Cancer | |  |  |
|  |  |  | Chronic result of accidents | |  |  |
|  |  |  | Nasal polypus | |  |  |
|  |  |  | Surgery | |  |  |
|  |  |  | Thyroid disease | |  |  |
|  |  |  | Tumor | |  |  |
|  |  |  | Liver cirrhosis | |  |  |
|  |  |  | Lung problem | |  |  |
|  |  |  | Eye problem | |  |  |
|  |  |  | Fever | |  |  |
|  |  |  | Chikungunya | |  |  |
|  |  |  | Birth complications (pre/post/pregnancy) | |  |  |
|  |  |  | Stomach pain | |  |  |
|  |  |  | Weakness | |  |  |
|  |  |  | Arsenic | |  |  |
|  |  |  | Allergy | |  |  |
|  |  |  | Dysentery | |  |  |
|  |  |  | Chickenpox | |  |  |
|  |  |  | Measles | |  |  |
|  |  |  | Others  (Please specify_________) | | 555 |  |
| 77 | Where did you seek treatment for this illness? (If you went to more than one place to seek treatment for this illness, where did you go first?) | | CHCP in Community Clinic | |  |  |
|  |  |  | Doctor in Upazilla Health Complex | |  |  |
|  |  |  | Doctor in district level health complex | |  |  |
|  |  |  | Doctor in union health and family welfare Centre | |  |  |
|  |  |  | SACMO in UHC | |  |  |
|  |  |  | Dental Surgeon | |  |  |
|  |  |  | Doctor in specialized health complex | |  |  |
|  |  |  | Private medical practitioner | |  |  |
|  |  |  | Paramedic | |  |  |
|  |  |  | Village Doctor | |  |  |
|  |  |  | Health assistant | |  |  |
|  |  |  | Pharmacist | |  |  |
|  |  |  | Drug seller | |  |  |
|  |  |  | NGO medical facility | |  |  |
|  |  |  | Homeopathy doctor | |  |  |
|  |  |  | Others  (Please specify_________) | | 555 |  |
|  |  |  | Don’t know | | -99 |  |
| 78 | Did they take any antibiotics for this illness? | | Yes | | 1 |  |
|  |  |  | No | | 2 | **→ 85** |
|  |  |  | Don’t know | | -99 | **→ 85** |
| 79 | Where did they get the antibiotics from: 1) given them by a family member or a friend, 2) bought for them (from a pharmacy for example), or 3) given to them for free by a healthcare provider?  (Multiple Answers are acceptable) | | Given by family members or friends | | 1 | **→ 85** |
|  |  |  | Bought | | 2 |  |
|  |  |  | Free from provider | | 3 | **→ 82** |
|  |  |  | Don’t know | | 4 | **→ 85** |
| 80 | Where did they or a family member buy the antibiotics from? | | Pharmacy | |  |  |
|  |  |  | Local grocery shop | |  |  |
|  |  |  | Private medical practitioner | |  |  |
|  |  |  | Paramedic clinic | |  |  |
|  |  |  | Village Doctor Clinic | |  |  |
|  |  |  | NGO medical facility | |  |  |
|  |  |  | Other (please specify) | | 555 |  |
|  |  |  | Don’t know | | -99 |  |
| 80_a | Usually community clinic, upazilla health complex, District health complex, SACMO in UHC provide free medicine to the patients.  As they have taken treatment from Community Clinic/UHC/District Hospital/SACMO in UHC and Purchase antibiotic medicine from somewhere else  What are the reasons to buy antibiotic medicines from somewhere else? | | Doctor from the health facility advised to purchase medicine from outside | |  | If the answer to question **Q77** is any of the options among 1, 2, 3, 4, 5 and if the answer to question **Q79** is 2 or multiple options, where option 2 is included then the questioner will ask **Q80** and then **Q80_a** will apply. |
|  |  |  | Advised medicine from the doctor is not available at the health facility | |  |  |
|  |  |  | The medicine was not stocked in the health center | |  |  |
|  |  |  | The full course of medicine was not given by the health facility | |  |  |
|  |  |  | Others  (Please specify_________) | | 555 |  |
| 81 | When they or a family member (on their behalf) bought the antibiotics did you they use a prescription? | | Yes | | 1 |  |
|  |  |  | No | | 2 |  |
|  |  |  | Don’t know | | -99 |  |
| 82 | Which health care provider gave them or a family member the antibiotics for them? | | CHCP in Community Clinic | |  |  |
|  |  |  | Doctor in Upazilla Health Complex | |  |  |
|  |  |  | Doctor in district level health complex | |  |  |
|  |  |  | Doctor in union health and family welfare Centre | |  |  |
|  |  |  | SACMO in UHC | |  |  |
|  |  |  | Dental Surgeon | |  |  |
|  |  |  | Doctor in specialized health complex | |  |  |
|  |  |  | Private medical practitioner | |  |  |
|  |  |  | Paramedic | |  |  |
|  |  |  | Village Doctor | |  |  |
|  |  |  | Health assistant | |  |  |
|  |  |  | Pharmacist | |  |  |
|  |  |  | Drug seller | |  |  |
|  |  |  | NGO medical facility | | 14 |  |
|  |  |  | Homeopathy doctor | | 15 |  |
|  |  |  | Other (please specify) | | 555 |  |
|  |  |  | Don’t know | | -99 |  |
| 83 | Did the provider give a prescription for the antibiotics? | | Yes | | 1 |  |
|  |  |  | No | | 2 |  |
|  |  |  | Don’t know | | -99 |  |
| 84 | Did they take all the antibiotics they got? | | Yes | | 1 |  |
|  |  |  | No | | 2 |  |
|  |  |  | Don’t know | | -99 |  |
| ***If there are multiple family members aged under 15, repeat this section for each of the family members from Q73.*** | | | | | | |
| 84_n_a | | Have you heard that all medicine known as antibiotics will now come in packs with a red label/marking and the word antibiotics on, warning to only take them if prescribed by a registered physician? | | Yes | 1 | |
|  |  |  |  | No | 2 | |
|  |  |  |  | Don’t Know | -99 | |
| 84_n_b | | ARE you aware that CHCPs will no longer be able to prescribe Antibiotics?  Or  “Soon you will no longer be given antibiotics medicine from Community Clinics”-  Do you know anything about it? | | Yes | 1 | |
|  |  |  |  | No | 2 | |
|  |  |  |  | Don’t Know | -99 | |
|  | |  | |  | | |
| ***Observable indicators*** | | | | | | |
|  |  | |  | |  |  |
| 85 | Presence of handwashing facility with water and soap/alcohol gel/detergent (ash/mud/sand) at the home? | | Yes | | 1 |  |
|  |  |  | No | | 2 |  |
| 86 | Are animals able to access and drink from the same source of water used by the household to drink and cook with? | | Yes | | 1 |  |
|  |  |  | No | | 2 |  |
| 87 | If the household owns any animal, how animal faeces are dealt with in the household? | | Stored for Fertilizer | | 1 |  |
|  |  |  | Thrown away to nearby water source | | 2 |  |
|  |  |  | Use as Biogas | | 3 |  |
|  |  |  | Use as Fuel | | 4 |  |
|  |  |  | Buried in Ground | | 5 |  |
|  |  |  | Other Uses (Please specify)  ________________________ | | 555 |  |
| 88 | Do you think handwashing with soap/gel/detergent (ash/mud/sand) etc could reduce the possibility of getting diseases and thus reduce the possibility of requiring antibiotics? | | Yes | | 1 |  |
|  |  |  | No | | 0 |  |
|  |  |  | Don’t Know | | -99 |  |
| 89 | What types of drinking water you and your family usually drink? | | Boiled Water | |  |  |
|  |  |  | Supply Water | |  |  |
|  |  |  | Normal Filtered Water | |  |  |
|  |  |  | Purified Filtered water | |  |  |
|  |  |  | Deep Tubewell Water | |  |  |
|  |  |  | Normal Tubewell water | |  |  |
|  |  |  | Pond Water | |  |  |
|  |  |  | River Water | |  |  |
|  |  |  | Well Water | |  |  |
|  |  |  | Rain harvested water | | 1. 9 |  |
|  |  |  | Others (Please specify) | | 555 |  |
| 90 | What types of toilets usually do you and your family use? | | Modern Toilet with Flush facility | | 1 |  |
|  |  |  | Modern Toilet without Flush facility | | 2 |  |
|  |  |  | Slab Toilet | | 3 |  |
|  |  |  | Pit Toilet | | 4 |  |
|  |  |  | Open/hanging Toilet | | 5 |  |
|  |  |  | No arrangement/Open Space/ Bush | | 6 |  |
|  |  |  | Others (Please specify) | | 555 |  |

**Annex:**

| **Subdistrict (strata)** | **Code** | | **Union** | **Code** | **Community clinic (cluster)** | **Village** | **Household/ respondent id number** |
| --- | --- | --- | --- | --- | --- | --- | --- |
| Barura | 10 | | \| Aganagar union \| 01 \| \| --- \| --- \| \| Bhabanipur union \| 02 \| \| Khoshbash (north) union \| 03 \| \| Jhalam union \| 04 \| \| Chittadda union \| 05 \| \| Shilmuri (south) union \| 06 \| \| Shilmuri (north) union \| 07 \| \| Galimpur union \| 08 \| \| Shakpur union \| 09 \| \| Vaukosar union \| 10 \| \| Chandla union \| 11 \| \| Adda union \| 12 \| \| Adra union \| 13 \| | | \| Gobindapur community clinic \| \| --- \| \| Arifpur community clinic \| \| Falkamuri community clinic \| \| Noagao community clinic \| \| Modho laksipur community clinic \| \| Jaynagar community clinic \| \| Gohaliya community clinic, barura \| \| Ghosapa community clinic \| \| Mukgao community clinic, barura \| \| Arai community clinic \| \| Uttar laksipur community clinic \| \| Kalora community clinic \| \| Baraipur community clinic \| \| Mahidpur cc \| \| Narinda community clinic \| | (name)  *(Please write down the village name)* | Hh_id |
| Brahman para | | 20 | \| Shidlai union \| 01 \| \| --- \| --- \| \| Chandla union \| 02 \| \| Shoshidol union \| 03 \| \| Dulalpur (2) union \| 04 \| \| Brahmanpara sadar union \| 05 \| \| Sahebabad union \| 06 \| \| Malapara union \| 07 \| \| Madhabpur union \| 08 \| | | \| Dulalpur community clinic \| \| --- \| \| Sahababad community clinic \| \| Deush community clinic \| \| Asadnagar community clinic \| \| Berakhala community clinic \| \| Kalpabas community clinic \| \| Manura community clinic \| \| Tetavumi community clinic \| \| Makimpur community clinic \| \| Uttar candala community clinic \| | (name)  *(Please write down the village name)* | Hh_id |
| Burichang | | 30 | \| Maynamati union \| 01 \| \| --- \| --- \| \| Bharella union \| 02 \| \| Mokam union \| 03 \| \| Burichang sadar union \| 04 \| \| Bakshimul union \| 05 \| \| Pirzatrapur union \| 06 \| \| Sholonol union \| 07 \| \| Rajapur union \| 08 \| | | \| Panchkista community clinic \| \| --- \| \| Rampur community clinic \| \| Kalakachuya community clinic \| \| Agapara community clinic \| \| Kothanagar community clinic \| \| Abidpur community clinic \| \| Kakirchar community clinic \| \| Pachora community clinic \| \| Paschim jagatpur community clinic \| \| Kamar khara community clinic \| \| Haripur community clinic \| \| Pitabar community clinic \| \| Sinduriyapara community clinic \| | (name)  *(Please write down the village name)* | Hh_id |
| Daudkandi | | 40 | \| Daulatpur union \| 01 \| \| --- \| --- \| \| Daudkandi (north) union \| 02 \| \| Elliotganj (north) union \| 03 \| \| Elliotganj (south) union \| 04 \| \| Jinglatoli union \| 05 \| \| Sundolpur union \| 06 \| \| Gouripur union \| 07 \| \| Mohammadpur (east) union \| 08 \| \| Mohammadpur (west) union \| 09 \| \| Gowalmari union \| 10 \| \| Maruka union \| 11 \| \| Biteshwar union \| 12 \| \| Padua union \| 13 \| \| Panchgachia west union \| 14 \| \| Barpara union \| 15 \| | | \| Itakhola community clinic \| \| --- \| \| Pipiyakhandi community clinic \| \| Syadkhar kandi community clinic \| \| Paiker char community clinic \| \| Patanikandi community clinic \| \| Lakshipur community clinic \| \| Sundalpur community clinic \| \| Maruka community clinic \| \| Uttar bainagar community clinic \| \| Nuton hasnabad community clinic , daudkandi \| \| Nalchak community clinic \| | (name)  *(Please write down the village name)* | Hh_id |
| Homna | | 50 | \| Mathabhanga union \| 01 \| \| --- \| --- \| \| Ghagutia union \| 02 \| \| Asadpur union \| 03 \| \| Chanderchar union \| 04 \| \| Bhashania union \| 05 \| \| Nilkhi union \| 06 \| \| Gharmora union \| 07 \| \| Joypur union \| 08 \| \| Dulalpur union \| 09 \| | | \| Fater kandi community clinic \| \| --- \| \| Douloth pur community clinic \| \| Kondokar char community clinic \| \| Jaynagar community clinic \| \| Maijchar community clinic \| \| Munshi kandi community clinic \| \| Nager char community clinic \| \| Moddo kandi community clinic \| \| Sharifpur community clinic \| \| Sreemoddi community clinic \| \| Mohanpur community clinic \| \| Nilakhi community clinic \| | (name)  *(Please write down the village name)* | Hh_id |
| Latitude | |  | | | | | |
| Longitude | |  | | | | | |

# 5. Community dialogue sessions’ key messages

| **Table S5. Community dialogue sessions’ key messages** | | |
| --- | --- | --- |
| **Session no.** | **Session title** | **Key messages** |
| 1 | An introduction to COSTAR | - Introduction to COSTAR - Safeguarding - COVID-19 issues - Mapping Exercise |
| 2 | Microbes, antibiotics and antimicrobials | - Symptoms need to be checked by a professional. It’s not always obvious whether an infection is viral or bacterial. - Different illnesses in people and animals are caused by different microbes. - Different infections need different treatments. Treatments may include medicines like antibiotics. - Antibiotics are a common medicine that are used to treat bacterial infections. They come in different forms such as pills, injections, liquids, or creams for use outside the body. Health professionals will advise you which one to use. - Antibiotics save lives of humans and animals and make them feel better when taken correctly. - Only use antibiotics when prescribed by a certified health professional. Same message for humans, fish and animals. |
| 3 | Access to antibiotics | - Only use antibiotics when prescribed by a certified health professional. Same message for humans, fish and animals. - The symptoms of many illnesses can look the same, but they can be caused by different infections (bacteria, viruses, parasites or fungi). - Buying antibiotics without consulting a health professional could mean you are given the wrong medicine for your illness, too much or too little of the medicine. - Only use antibiotics when prescribed by a registered health provider to ensure the proper treatment of the infection and complete recovery. This will ensure they remain effective in the future. - Health professionals can conduct tests to find out which infection is making you/your animal ill so that they can prescribe the right medicine. - If you need to purchase antibiotics, always take your registered health provider’s prescription, and buy a complete course. - Expensive antibiotics are not necessarily better. - Always follow your health worker’s/vet’s advice on when and how to use antibiotics. If humans and animals are to get better from illnesses, they need to make sure the right medicines are taken for the right amount of time and in the right amount (dosage). - Symptoms need to be checked by a professional. It’s not always obvious whether an infection is viral or bacterial. |
| 4 | Safe utilization of antibiotics | - Always follow your health worker’s/vet’s advice on when and how to use antibiotics. If humans and animals are to get better from illnesses, they need to make sure the right medicines are taken for the right amount of time and in the right amount (dosage). - Never save antibiotics for later or share antibiotics with another person or animal, as this poses risks for you and others. - There should be no antibiotic leftover, but if there are any, they should be taken to the community health clinic and handed over to the health providers for proper use/disposal. - It is important not to use antibiotics prescribed for humans on animals and vice versa. This will ensure they remain effective in the future. - Do not throw the leftover or expired antibiotics down the toilet or in the open environment as they may harm the good bacteria. |
| 5 | Antimicrobial resistance and how antibiotic misuse drives AMR | - Antibiotics save lives of humans and animals and make them feel better when taken correctly. - Bacteria can learn how to ‘resist’ the effect of antibiotics and other medicines. This is called AMR. - AMR is very dangerous because infections that were easily treatable are killing once again because they have become drug-resistant infections, which means they can no longer be cured by modern medicine. - Every year there are more and more antibiotic-resistant infections. The problem of AMR will get worse over time. We need to take immediate action. - Antibiotic-resistant infections spread from one person to another person AND from animals to humans. - Antibiotic-resistant infections are harder to treat than infections that are not resistant to antibiotics. - There are many simple behaviours we can do to make AMR less likely to happen, and keep ourselves, our families, communities and animals healthy and productive. |
| 6 | Recap session | - Antibiotics save lives of humans and animals and make them feel better when taken correctly. - Only use antibiotics when prescribed by a certified health professional. (Same message for humans, fish and animals). - The symptoms of many illnesses can look the same, but they can be caused by different infections (bacteria, viruses, parasites or fungi). - Buying antibiotics without consulting a health professional could mean you are given the wrong medicine for your illness, too much or too little of the medicine. - Only use antibiotics when prescribed by a registered health provider to ensure the proper treatment of the infection and complete recovery. This will ensure they remain effective in the future. - If you need to purchase antibiotics, always take your registered health provider’s prescription, and buy a complete course. - Using the wrong medicine, in the wrong amount or for the wrong amount of time could cause AMR. - Symptoms need to be checked by a professional. It’s not always obvious whether an infection is viral or bacterial. - Care for a sick person by providing good easily digested food in small quantities, reducing fever by using warm bathing or fanning and giving anti-inflammatories increasing fluid intake enabling rest. |
| 7 | Staying healthy | - Using the wrong medicine, in the wrong amount or for the wrong amount of time could cause AMR. - Symptoms need to be checked by a professional. It’s not always obvious whether an infection is viral or bacterial. - Care for a sick person by providing good easily digested food in small quantities, reducing fever by using warm bathing or fanning and giving anti-inflammatories increasing fluid intake enabling rest. |
| 8 | AMR and animals | - Different infections need different treatments. Treatments may include medicines like antibiotics. - Antibiotics save lives of humans and animals and make them feel better when taken correctly. - Only use antibiotics when prescribed by a certified health professional. (Same message for humans, fish and animals). - The symptoms of many illnesses can look the same, but they can be caused by different infections (bacteria, viruses, parasites or fungi). - All infections, including resistant infections, can be spread between people, animals and the environment. - Faeces, food products, dirty water and contaminated soil (soil that has human and animal waste, or other types of pollution washed into it in large quantities) are some of the ways infections, and resistant infections, can move between people, animals and the environment. - Maintain the antibiotic withdrawal time and do not sell or slaughter your animal until the withdrawal period is over. Ask your vet when to sell or slaughter because the withdrawal period varies depending on the antibiotic group and the type of animal and animal product (e.g. meat/milk). - Meat or other produce (e.g. milk) from an animal that has recently been treated with an antibiotic can contain antibiotics. - Do not use antibiotics for growth promotion or to prevent diseases in healthy animals or fish. Instead provide animals and fish with fresh, healthy and nutritious food rather than adding antibiotics to their food and give newborn animals the colostrum from their mothers. - Only give antibiotics to animals and fish under veterinary supervision. Only use this medication if it has been prescribed by a licensed/registered veterinarian (or if unavailable call vet, or use paravet). - Do not use antibiotics for growth promotion or to prevent diseases in healthy animals or fish. Instead provide animals and fish with fresh, healthy and nutritious food rather than adding antibiotics to their food and give newborn animals the colostrum from their mothers. - Meat or other produce (e.g. milk) from an animal that has recently been treated with an antibiotic can contain antibiotics. - Maintain the antibiotic withdrawal time and do not sell or slaughter your animal until the withdrawal period is over. Ask your vet when to sell or slaughter because the withdrawal period varies depending on the antibiotic group and the type of animal and animal product (e.g. meat/milk). - Preventing cross-contamination between people, animals and the environment is an important way to stop infections and AMR spreading. |
| 9 | Humans, animals and the environment | - Using the wrong medicine, in the wrong amount or for the wrong amount of time could cause AMR. - Preventing cross-contamination between people, animals and the environment is an important way to stop infections and AMR spreading. - Ensure animals and fish are kept in areas where they are not crowded; this helps to minimize infections spreading and the need for antibiotics. - Build separate animal houses to reduce the risk of zoonotic infection or AMR spreading. |
| 10 | Recap | - The cost of AMR |
| 11 | Reflection, wrap up and moving forward | - Future plan and sustainability |

# References

1. Campbell M, Katikireddi SV, Hoffmann T, Armstrong R, Waters E, Craig P. TIDieR-PHP: a reporting guideline for population health and policy interventions. BMJ. 2018;361:k1079.

2. Malaria Consortium. A guide to implementing the Community Dialogue Approach. 2018.

3. Martin S, Rassi C, Antonio V, Graham K, Leitao J, King R, Jive E. Evaluating the feasibility and acceptability of a community dialogue intervention in the prevention and control of schistosomiasis in Nampula province, Mozambique. Plos One. 2021;16(8).

4. King R, Hicks J, Rassi C, Shafique M, Barua D, Bhowmik P, et al. A process for developing a sustainable and scalable approach to community engagement: community dialogue approach for addressing the drivers of antibiotic resistance in Bangladesh. BMC Public Health. 2020;20(1):950.

5. Malaria Consortium. The Community Dialogue Approach 2024 [Available from: <https://www.malariaconsortium.org/pages/the-community-dialogue-approach/the-community-dialogue-approach-expertise.htm>.

6. Walley J, Khan MA, Witter S, Haque R, Newell J, Wei X. Embedded health service development and research: why and how to do it (a ten-stage guide). Health Research Policy and Systems. 2018;16(1):67.
